# CDADC1 is a vertebrate-specific dCTP deaminase that metabolizes gemcitabine and decitabine to prevent cellular toxicity

**DOI:** 10.1073/pnas.2424409122

**Published:** 2025-06-12

**Authors:** Marcelo M. Rodriguez, Debashree Chatterjee, Johanna Guerry, Anne-Marie Patenaude, Charles C. H. Cohen, Therence Bois, Ariane Larouche, Silvana R. Ferreira, Thierry Bertomeu, Andrew Chatr-aryamontri, Li Zhang, Sylvie Mader, Corey Nislow, Guillaume St-Jean, Yvan Guindon, Astrid Zahn, Javier M. Di Noia

**Affiliations:** ^a^Institut de Recherches Cliniques de Montréal, Montréal, QC H2W 1R7, Canada; ^b^Molecular Biology Programs, Faculty of Medicine, Université de Montréal, Montréal, QC H3C 3J7, Canada; ^c^Chemogenix, Université de Montréal, Montréal, QC H3T 1J4, Canada; ^d^Institute for Research in Immunology and Cancer, Université de Montréal, Montréal, QC H3T 1J4, Canada; ^e^Faculty of Pharmaceutical Science, University of British Columbia, Vancouver, BC V6T 1Z3, Canada; ^f^Department de Pathology and Microbiology, Faculty of Veterinary Medicine, Université de Montréal, Saint-Hyacinthe, QC J2S 2M2, Canada; ^g^Department of Chemistry, Faculty of Arts and Sciences, Université de Montréal, Montréal, QC H3C 3J7, Canada; ^h^Department of Medicine, Faculty of Medicine, Université de Montréal, Montréal, QC H3C 3J7, Canada

**Keywords:** cytidine deaminase, nucleotide metabolism, dCTP deaminase, gemcitabine, decitabine

## Abstract

Cancer therapy is limited by resistance to standard-of-care chemotherapeutic and/or by treatment-associated toxicity. Identifying molecular mechanisms that modulate cellular toxicity is crucial for enhancing treatment efficacy. We characterize CDADC1, a vertebrate-specific orphan enzyme, as an unprecedented eukaryotic dCTP deaminase. CDADC1 catalyzes the conversion of dCTP into dUTP. While bacteria use this activity to sustain proliferation, CDADC1 evolved independently and is not required for mammalian cell proliferation, as demonstrated in cell lines and by the normal growth and standard lifespan of Cdadc1-deficient mice. However, we uncover a role of CDADC1 in metabolizing nucleotide analogs gemcitabine and decitabine. Gain- and loss-of-function assays in cancer cell lines, along with ectopic mouse models of pancreatic cancer, show that CDADC1 reduces these drugs’ efficacy. By the same token, *Cdadc1^−/−^* mice are hypersensitive to gemcitabine. Mechanistically, CDADC1 deaminates the active triphosphate form of gemcitabine and decitabine, rendering them susceptible to inactivation by deoxyuridine triphosphatase. In contrast, the dCMP deaminase DCTD contributes to cell proliferation and promotes gemcitabine and decitabine toxicity. Thus, CDADC1 underpins a previously unrecognized mechanism of intrinsic chemoresistance in cancer cells and has a nonredundant role in protecting from gemcitabine toxicity. CDADC1 reveals a clinically relevant metabolic pathway that might be exploited to enhance the efficacy of deoxycytidine analogs but calls for assessing CDADC1 status to avoid lethal toxicities.

## Significance

We identify CDADC1, an orphan enzyme unique to vertebrates, as a dCTP deaminase that produces dUTP. This reaction was previously reported only in some bacteria, where it supports de novo synthesis of dTTP. However, we rule out a main role of CDADC1 in dTTP biosynthesis, cell proliferation, or mouse viability. Nonetheless, CDADC1’s activity implies a role for dUTP in vertebrate cells, challenging the prevailing view of dUTP solely as a toxic byproduct. In addition, CDADC1 metabolizes and inactivates the nucleotide analogs gemcitabine and decitabine. CDADC1 deficiency in cancer cells increases these drugs’ efficacy but also protects mice from lethal gemcitabine-induced toxicity. We reveal a biochemical pathway in vertebrate nucleotide metabolism with clinical implications for optimizing gemcitabine and decitabine treatment.

De novo biosynthesis of three deoxynucleotide triphosphates (dATP, dGTP, and dCTP) occurs via reduction of their ribonucleotide diphosphate precursors by ribonucleotide reductase (1). In contrast, dTTP synthesis requires a dedicated enzyme, thymidylate synthase (TYMS), which converts deoxyuridine monophosphate (dUMP) to deoxythymidine monophosphate, subsequently phosphorylated to dTTP (1, 2). This dUMP dependency leads to production of dUTP, considered an unwanted byproduct that competes with dTTP during replication (1, 3). Excessive uracil in DNA can overwhelm repair mechanisms, leading to genotoxic DNA breaks (3, 4). To counter uracil incorporation, proliferating cells express deoxyuridine triphosphatase (DUT), which hydrolyzes dUTP to dUMP, and uracil-DNA glycosylases (1, 5).

The reaction catalyzed by TYMS is rate-limiting for cell proliferation (2). The substrate of TYMS can be generated either via ribonucleotide reductase (UDP to dUDP to dUMP) or through the activity of deoxycytidylate deaminases (6). Gram-negative bacteria like *Escherichia coli*, produce dUMP via dcd, a dCTP deaminase that converts dCTP to dUTP, which DUT converts to dUMP (7, 8). Some archaeans have bifunctional dcd-dut enzymes for this purpose (9). No *dcd* ortholog or dCTP deaminase activity has been reported in eukaryotic species (7, 10, 11). Eukaryotes rely on a deoxycytidine monophosphate (dCMP) deaminase, encoded by *DCTD* in vertebrates, which directly produces dUMP. While alternative sources exist, DCTD is often a major source of dUMP for dTTP biosynthesis in human cell lines (7, 11, 12).

The genotoxic properties of nucleotide analogs that interfere with DNA synthesis have been harnessed for cancer treatment, including several deoxycytidine (dC) analogs (13, 14). These compounds are administered as nucleosides and rely on active import and intracellular conversion to their triphosphate form to exert their cytotoxic effects. Gemcitabine (2′,2′-difluoro-2′-deoxycytidine, dFdC) is one of the most widely used, indicated for pancreatic, breast, lung, and ovarian cancer (14). Decitabine (5-aza-2′-deoxycytidine, 5-aza-dC) is indicated for myelodysplastic syndrome and leukemia (15). Both dFdCTP and 5-aza-dCTP are incorporated during DNA replication. Gemcitabine acts as a chain terminator, causing DNA damage, proliferation arrest, and cell death (16). Decitabine inhibits DNA methyltransferases, enabling reexpression of silenced genes detrimental to cancer cell survival (17).

Current clinical challenges for dC analogs include intrinsic or acquired resistance and off-target toxicity, which can be modulated by nucleotide metabolism enzymes (13, 18). Notably, deoxycytidine kinase (DCK) initiates the activation of dC analogs by generating the monophosphate precursor (19, 20). Two members of the Zn-dependent deaminase family are known to metabolize dC analogs in vertebrate cells: DCTD and cytidine deaminase (CDA). DCTD promotes gemcitabine and decitabine toxicity by mechanisms that are not fully elucidated (21, 22). In contrast, CDA causes resistance by converting dC analogs to their uracil derivatives (e.g., dFdC to dFdU), which are poor substrates for DCK (18, 20). Importantly, a fraction of patients displaying low serum CDA activity or carrying certain *CDA* polymorphisms can suffer from severe toxicity upon dC analog treatment (13, 18, 23, 24). CDA polymorphisms can only explain a proportion of the interindividual variation (13, 18, 23, 24), suggesting the presence of additional factors preventing toxicity.

Here, we identify the orphan member of the Zn-dependent deaminase family, cytidine and dCMP deaminase domain containing 1 (CDADC1) (25), as a vertebrate-specific dCTP deaminase, that catalyzes the direct conversion of dCTP into dUTP. CDADC1 is dispensable for cell growth, and *Cdadc1^−/−^* mice have normal lifespan and no overt phenotype. On the other hand, we demonstrate that endogenous CDADC1 confers substantial resistance to gemcitabine and decitabine in cancer cells. Notably, *Cdadc1^−/−^* mice are extremely sensitive to gemcitabine, revealing also a major role for CDADC1 in preventing therapy-associated toxicity. Mechanistically, CDADC1 deaminates the active dC analog triphosphate metabolites, generating uracil-like derivatives that can be hydrolyzed by DUT. CDADC1’s activity not only implies a function for dUTP vertebrate cells but also reveals a previously unrecognized pathway nucleotide metabolism affecting dC analogs, which is likely to have clinical relevance.

## Results

### CDADC1 Is a dCTP Deaminase.

**Fig. 1.**
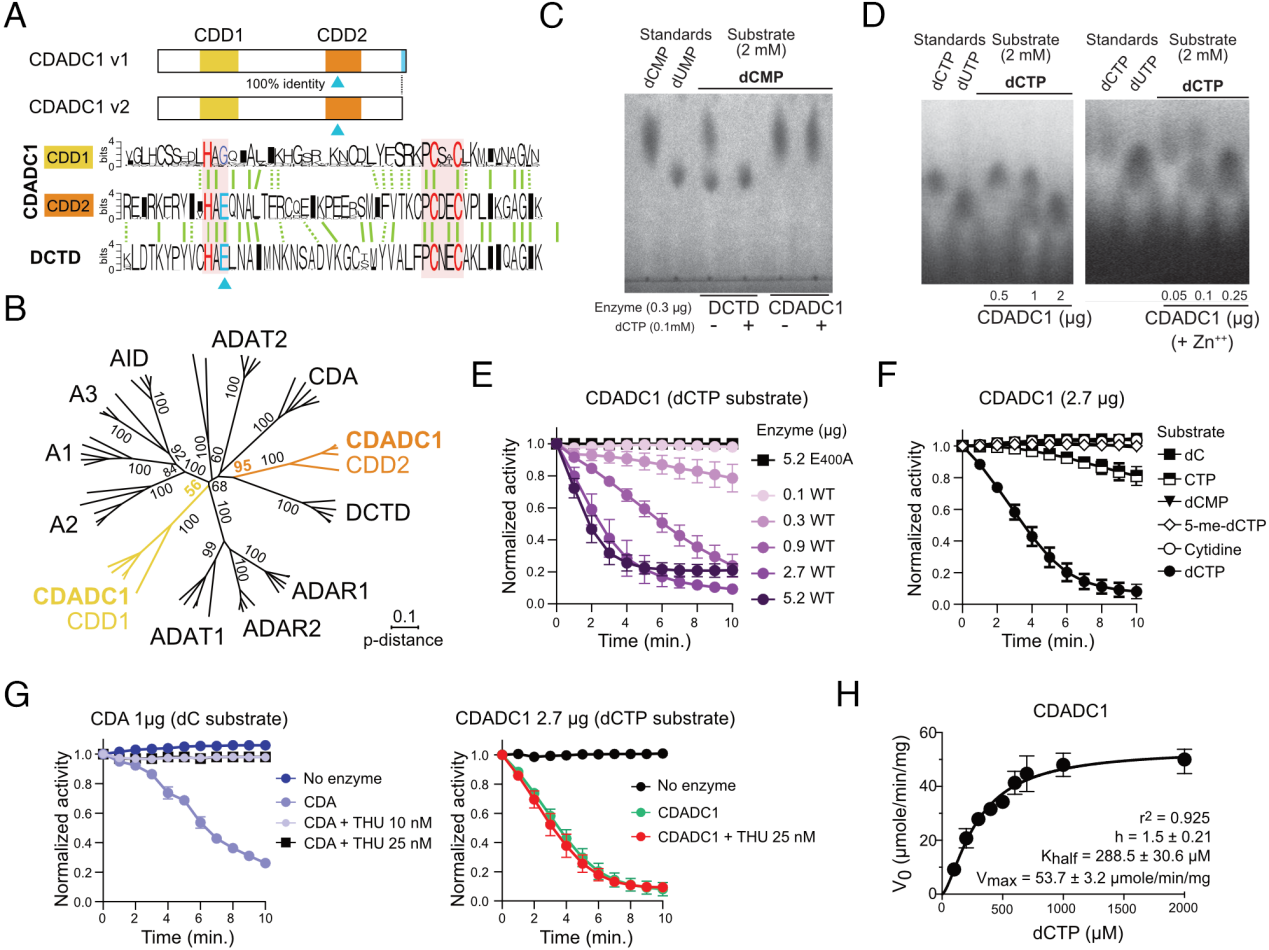
CDADC1 has dCTP deaminase activity. (*A*) Scheme of human CDADC1 isoforms (v1, v2) highlighting Zn-dependent deaminase domains (CDD1 and CDD2) and the C-terminal tail (light blue) of v1. Sequence logos of the CDD1, CDD2, and DCTD deaminase domains generated from sequence alignments from 18 species, highlighting bipartite deaminase motifs (red shading), Zn^++^-coordinating (red), and catalytic residue (blue triangle). Vertical lines represent identities (solid) or similarities (dotted). (*B*) Unrooted neighbor-joining tree based on aligned Zn-coordination motif ±10 residues from vertebrate deaminase classes. Bootstrap support is given as proportion of 2000 replicates (*C*) TLC of reactions with recombinant enzymes, dCMP as substrate, and the DCTD allosteric activator dCTP after 15 min at 37˚C. Standards (10 µg each) were run in parallel. (*D*) As in C) for recombinant CDADC1 with dCTP as substrate. CDADC1 used on the right-hand plate was supplemented with Zn^++^ throughout the purification procedure. (*E*) CDADC1 dose–response activity on dCTP, monitored by decrease in absorbance at 290 nm. Mutation E_400_A at the predicted CDD2 catalytic residue. (*F*) CDADC1 substrate specificity assayed as in (*E*), on various nucleotides and nucleosides. (*G*) Effect of preincubating recombinant CDA or CDADC1 with tetrahydrouridine (THU) for 1 min before monitoring deamination as in (*E*). (*E*–*G*) Normalized mean ± SEM from three reactions with independent protein preparations. (*H*) CDADC1 initial velocity (V_0_) as a function of dCTP substrate concentration in reactions with 200 ng recombinant enzyme. Mean ± SD from three reactions with independent protein preparations.

CDADC1 contains two Zn-dependent cytidine deaminase domains, CDD1 and CDD2 (Fig. 1*A*) (25). CDD1 lacks the glutamate typically involved in catalysis (26, 27), suggesting only CDD2 is active (Fig. 1*A*). Sequence alignment revealed no clear ancestry for CDD1 but showed that CDD2 shares a common ancestor with DCTD (Fig. 1*B*).

We purified recombinant CDADC1 and DCTD from *E. coli* (*SI Appendix*, Fig. S1*A*) and used thin-layer chromatography (TLC) to monitor deamination activity. As expected, DCTD converted dCMP to dUMP, with activity enhanced by the allosteric activator dCTP (Fig. 1*C*). CDADC1 showed no activity on dCMP (Fig. 1*C*) but was able to deaminate dCTP to dUTP (Fig. 1*D*). This activity increased when Zn^++^ was supplemented during purification (Fig. 1*D*), consistent with Zn^++^ dependence of this enzyme family. No activity was detected on CTP, cytidine, or deoxycytidine by TLC (*SI Appendix*, Fig. S1*B*).

To confirm these results with higher sensitivity, we used an assay based on differential extinction coefficients of cytosine and uracil, detecting deamination as decreased absorbance at 290 nm (see detailed method in *SI Appendix*). Activity on dCTP was abolished by mutating glutamate in position 400 to alanine in CDD2 (Fig. 1*E*). CDADC1 showed no activity on dCMP, cytidine, or dC (Fig. 1*F* and *SI Appendix*, Fig. S1*C*). Unlike CDA and DCTD, which tolerate 5′ methyl groups (28, 29), CDADC1 did not visibly deaminate 5-methyl-dCTP (Fig. 1*F*). CDADC1 was also insensitive to the CDA inhibitor tetrahydrouridine (THU) (30) (Fig. 1*G*). On the other hand, this assay detected CDADC1 activity on CTP, albeit substantially lower than on dCTP (Fig. 1*F* and *SI Appendix*, Fig. S1*D*). These findings suggest that the triphosphate stabilizes the substrate, whereas a 2′OH or modifications at position 5 of the pyrimidine ring create steric hindrance.

CDADC1 kinetics fit slightly better to a sigmoidal (r^2^ = 0.924) than Michaelis–Menten (r^2^ = 0.90) model, yielding Km and Vmax for dCTP that were comparable to DCTD’s for dCMP in the presence of the allosteric activator (Fig. 1*H* and *SI Appendix*, Fig. S1*E*), supporting dCTP as a natural substrate.

CDADC1 belongs to the same family as the DNA deaminases AID/APOBECs (Fig. 1*B*). We tested whether CDADC1 could deaminate DNA by asking whether it could induce mutations in bacteria. We expressed CDADC1 in uracil-DNA glycosylase-deficient *E. coli ung-1*, which enhances sensitivity by preventing dU excision (31), monitoring the frequency of RpoB mutations conferring rifampicin resistance. This assay detects the activity of all AID/APOBEC enzymes despite their different 5′ and 3′ nucleotide preferences (31, 32). While the positive control AID increased rifampicin-resistant colonies >10-fold over controls (empty vector and catalytically inactive AID E_58_A), CDADC1 had no effect (*SI Appendix*, Fig. S1*F*).

In conclusion, CDADC1 is a cytosine triphosphate nucleotides-specific deaminase, preferentially acting on dCTP.

### CDADC1 Is Dispensable for Cell Proliferation.

**Fig. 2.**
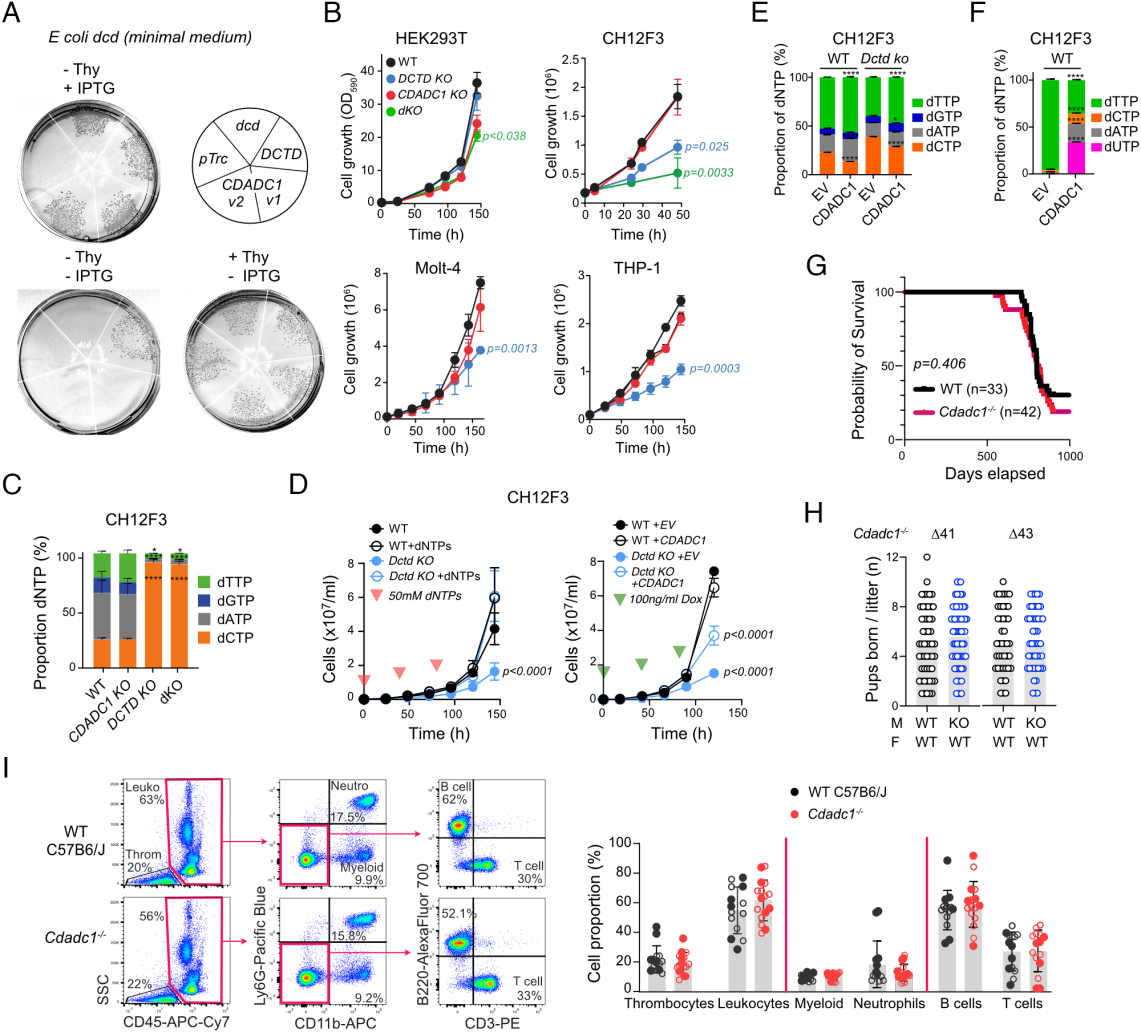
CDADC1 is dispensable for cell proliferation and in mice. (*A*) Growth of *E. coli dcd* complemented with IPTG-inducible pTrcHis empty vector (pTrc) or encoding dcd, human DCTD, or CDADC1 isoforms in minimal medium. Thy, thymidine. (*B*) Growth of isogenic WT, *CDADC1* KO, and *DCTD* KO cell lines, and double *CDADC1- DCTD*-deficient clones (dKO) in HEK293T and CH12F3 cells. Mean ± SEM for 3 (HEK293T, CH12F3), 5 (Molt-4), or 2 (THP-1) experiments, with two independent clones per genotype. Significant *P*-values from 2-way ANOVA with Dunnett multiple comparison test. (*C*) Relative proportion of dNTPs by LC–MS/MS in CH12F3 cells. Mean + SEM of five biological replicates. *P*-values (****P < 0.0001, *P < 0.05) for significant differences versus WT from 2-way ANOVA with Dunnett multiple comparison test. (*D*) Growth of CH12F3 WT and *Dctd* KO cells supplemented with dNTPs (mean ± SEM of 2 experiments, 2 biological replicates each) or expressing either doxycycline (dox)-inducible pSLIK empty vector (EV) or CDADC1v1 (mean ± SEM of 3 experiments). Significant P-values from 2-way ANOVA with Dunnett multiple comparison test. (*E*) Relative proportion of dNTPs by LC–MS/MS in cells from (*D*), as in (*C*). (*F*) Relative proportion of dNTPs by ion pairing LC–MS/MS in CH12F3 cells expressing pSLIK (EV) or CDADC1v1. This method detects dUTP but cannot resolve dGTP. (*E* and *F*) Mean + SEM of four biological replicates per genotype. *P*-values (****P < 0.0001, *P < 0.05) for significant differences versus EV in each genotype from 2-way ANOVA with Bonferroni’s multiple comparisons test. (*G*) Kaplan–Meier survival plot *Cdadc1^−/−^* mice (two *Cdadc1^−/−^* lines, Δ41 and Δ43, are plotted together) versus WT littermates. *P*-value by the long-rank test. (*H*) Number of pups per litter comparing WT and *Cdadc1^−/−^* male littermates bred with WT females. Compilation of all litters produced by 9 (∆41) or 8 (∆43) breeding pairs in 1 y. (*I*) Representative flow cytometry and plot of mean (bars) and individual mice (symbols) proportions of blood leukocyte populations determined from the indicated gates, distinguishing male (filled) and female mice.

To confirm the substrate specificity of CDADC1 we tested whether it could functionally compensate *E. coli* dCTP deaminase. We used *E. coli* BW1105 (dcd-12::kan) (8), which we used to purify recombinant CDADC1 to avoid contamination with the bacterial activity. As expected, BW1105 could not grow in minimal medium without thymidine supplementation or dcd reintroduction (Fig. 2*A*). Expression of human DCTD restored growth, likely by supplying dUMP. Despite lacking dCMP activity, both human CDADC1 isoforms complemented the thymidine auxotrophy (Fig. 2*A* and *SI Appendix*, Fig. S1 *G* and *H*).

Like *DCTD*, *CDADC1* exhibits characteristics of a housekeeping gene (33), with stable expression across human tissues and cell lines. CDADC1 is expressed at lower levels than DCTD but comparable to *CDA* and *TYMS* (*SI Appendix*, Fig. S2 *A*–*D*). Unlike DCTD and TYMS, CDADC1 is not upregulated in highly proliferative cells (*SI Appendix*, Fig. S2*B*). While it is accepted that eukaryotes lack a dCTP deaminase-dependent dTTP biosynthesis pathway for proliferation, relying instead on DCTD (*SI Appendix*, Fig. S1*H*) (7), the biochemical activity of CDADC1 prompted the question of whether it contributed to proliferation.

Using CRISPR-Cas9 we deleted *CDADC1* in HEK293T and two human leukemia cell lines, which showed the highest expression among various cell types (*SI Appendix*, Fig. S2 *C* and *D*). We also ablated *Cdadc1* in the mouse B cell lymphoma line CH12F3. Western blot after immunoprecipitation confirmed the absence of CDADC1 protein in HEK293T and CH12F3 cells (*SI Appendix*, Fig. S3*A*). For comparison, we inactivated *DCTD* (*SI Appendix*, Fig. S3*B*). DCTD deficiency did not affect HEK293T cells but significantly reduced cell growth of CH12F3, Molt-4, and THP-1 cells, revealing their dependency on the deamination-dependent dTTP biosynthesis pathway (Fig. 2*B*). CDADC1 deficiency had no significant impact on proliferation in any cell line (Fig. 2*B*).

The combined CDADC1/DCTD deficiency slightly reduced growth in HEK293T and CH12F3, compared to DCTD alone (Fig. 2*B*). While this could be interpreted as CDADC1 providing a partial backup for DCTD, several findings suggested this was not the case. First, unlike Dctd deficiency, which caused dNTP imbalances in CH12F3 cells, notably a large dCTP increase (34, 35), *Cdadc1* KO had no effect on dNTP or NTP pools, alone or in combination with *Dctd* KO, as assessed by LC–MS/MS (Fig. 2*C* and *SI Appendix*, Fig. S3*C*). Second, while CDADC1 overexpression in CH12F3 *Dctd* KO partially rescued growth, it was less effective than dNTP supplementation despite high expression levels (Fig. 2*D* and *SI Appendix*, Fig. S3*D*). Third, CDADC1 overexpression modestly altered dNTP proportions, increasing dTTP and dUTP while reducing dCTP, consistent with its biochemical activity, but failed to restore dNTP balance (Fig. 2 *E* and *F*). Fourth, unlike DCTD, CDADC1 was not inhibited by dTTP despite having a conserved allosteric pocket (*SI Appendix*, Fig. S3 *E* and *F*). Last, we considered whether CDADC1 might support cell proliferation under stress conditions. CDADC1 deficiency did not cause any additional defect on cell growth in conditions of starvation or chronic hypoxia (*SI Appendix*, Fig. S3*G*).

We conclude that endogenous CDADC1 has minimal impact on dNTP pools and is dispensable for cell proliferation in vitro. While overexpressed CDADC1 generates dUTP that could be used for limited dTTP biosynthesis, this is unlikely to be its primary physiological role.

### CDADC1 Is Dispensable for Mouse Development or Normal Lifespan.

We generated Cdadc1-deficient mice using a CRISPR-Cas9 deletion approach (*SI Appendix*, Fig. S4 *A* and *B*). *Cdadc1^−/−^* mice showed no difference in lifespan compared to WT (Fig. 2*G* and *SI Appendix*, Fig. S4*C*) and exhibited no physical or health abnormalities, either during life or at necropsy. Although *Cdadc1* is most highly expressed in the testis (*SI Appendix*, Fig. S2 *A* and *B*) and regulated during spermatogenesis (25), male *Cdadc1^−/−^* mice remained fertile (Fig. 2*H*). Additionally, the mice showed normal blood leukocyte populations, suggesting that hematopoiesis, which depends on rapid cell proliferation, was unaffected in *Cdadc1^−/−^* mice (Fig. 2*I*). Thus, CDADC1 is dispensable for mouse development or proliferation in vertebrate tissues.

### CDADC1 Confers Resistance to Gemcitabine and Decitabine.

Having established that CDADC1 deaminated dCTP, we asked whether it could metabolize dC analogs, which are converted to triphosphates inside cells.

**Fig. 3.**
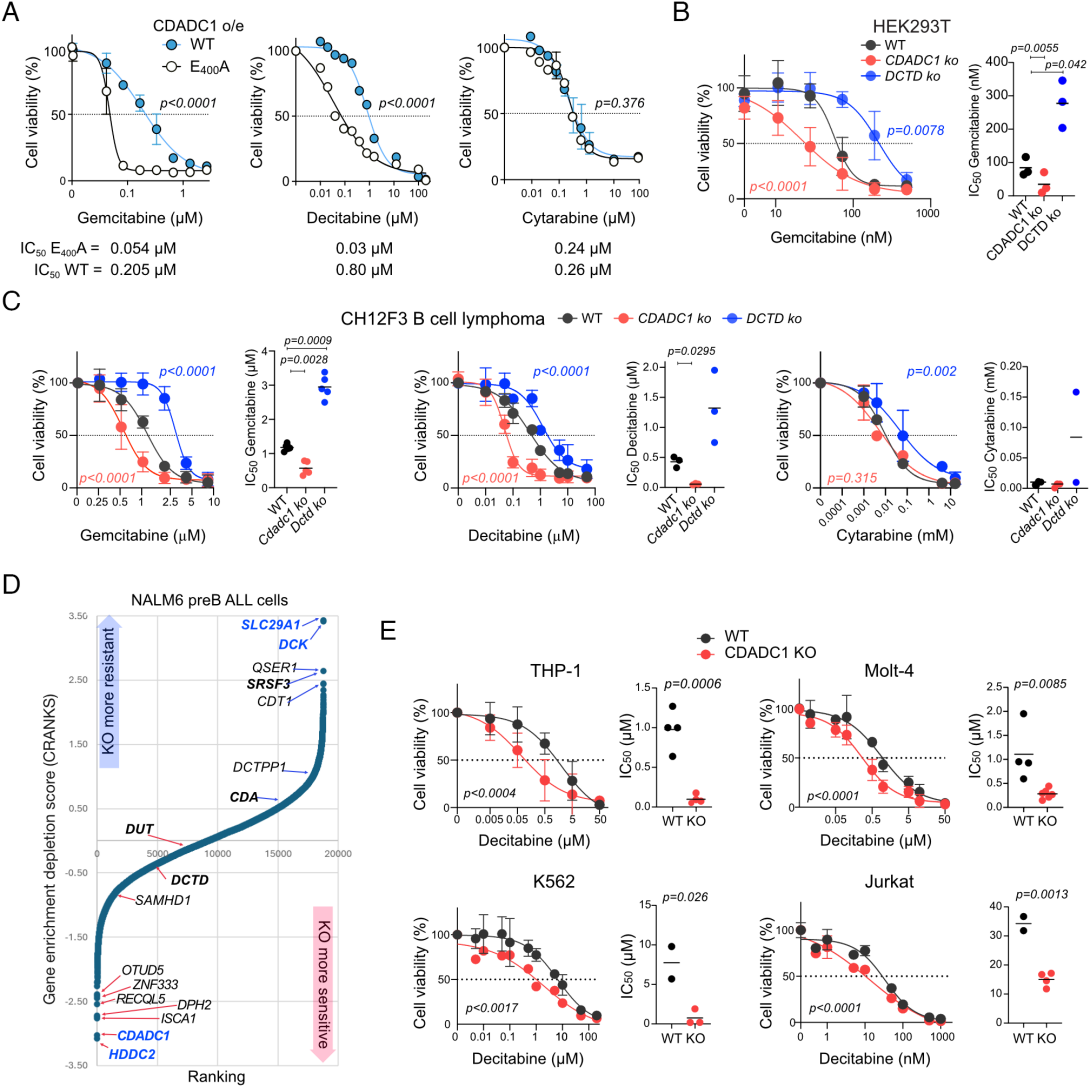
CDADC1 mediates resistance to gemcitabine and decitabine. (*A*) Relative cell viability of HEK293T cells expressing CDADC1v1 WT or E_400_A grown for 72 h over a range of dC analog concentration. Mean ± SD of three independent experiments (2 for decitabine). *P*-values from the 2-way ANOVA test. (*B*) Relative cell viability of HEK293T cells grown for 72 h in gemcitabine. Curves show mean ± SD from three independent experiments, with *P*-values from two-way ANOVA with Tukey’s multiple comparison. The scatter plot shows IC_50_ for biological replicates with significant *P*-values from two-way ANOVA with Bonferroni’s posttest. (*C*) Relative cell viability of CH12F3 cells grown in the presence of dC analogs. Analyzed and plotted as in (*B*) for 5 (gemcitabine) or 3 (decitabine, cytarabine) experiments. (*D*) Rank plot of genome-wide chemical genetic screen in NALM-6 cells treated with gemcitabine. Selected genes are indicated (blue = FDR < 0.05; bold = genes known to modulate gemcitabine sensitivity). (*E*) Relative cell viability of WT and *Cdadc1* KO human leukemia cells grown with decitabine for 72 h. Curves show mean ± SD from four independent experiments with two independent KO clones (THP-1, Molt-4); or from two experiments with 2 (Jurkat) or 3 (K562) independent KO clones. *P*-values from the two-way ANOVA test. Scatter plots show IC_50_ for biological replicates with *P*-values from the unpaired two-tailed *t* test.

HEK293T cells overexpressing human CDADC1 were more resistant to gemcitabine and decitabine than cells expressing the catalytically inactive CDADC1 E_400_A variant (Fig. 3*A*, S5A). Overexpression of active CDADC1 did not change sensitivity to cytarabine (Fig. 3*A*), a cytosine arabinoside used against leukemia and lymphoma and has a 2′OH (36), aligning with CDADC1’s biochemical preference for 2′-deoxynucleotides.

To determine whether endogenous CDADC1 modulated dC-analog response, we performed loss-of-function experiments. *CDADC1* KO HEK293T cells were ~threefold more sensitivity to gemcitabine than WT, whereas DCTD deletion increased resistance (Fig. 3*B*). Similarly, *Cdadc1* KO CH12F3 cells were sensitive to gemcitabine and decitabine, but not cytarabine, while Dctd deficiency enhanced resistance to all drugs (Fig. 3*C*).

For unbiased confirmation of the effect of CDADC1 in comparison with known cellular determinants of dC-analog resistance, we performed a genome-wide CRISPR/Cas9 KO chemogenomic screen in the pre-B acute lymphocytic leukemia NALM-6 cell line, grown in the presence of gemcitabine at a dosage that partially inhibited cell growth to identify genes that, when inactivated, revealed determinants of sensitivity and resistance (Dataset S1). The screen identified expected resistance factors, such as DCK and nucleoside transport SLC29A1, and sensitizers like HDDC2, a 5′-deoxynucleotidase (Fig. 3*D*). Among sensitizers, *CDADC1* ranked second-lowest in fitness score (CRANKS), indicating a meaningful contribution to gemcitabine sensitivity (Fig. 3*D*).

Given consistent CDADC1 expression across cancer types but with higher levels in leukemia (*SI Appendix*, Figs. S2*D* and S5 *B* and *C*), we compared decitabine sensitivity of WT and CDADC1-deficient cell lines from human T-cell acute lymphoblastic leukemia (Molt-4 and Jurkat), acute myeloid leukemia (THP-1), and chronic myeloid leukemia (K562). In each case, *CDADC1* KO cells were ~threefold more sensitive, independent of baseline drug sensitivity (Fig. 3*E*). Cytarabine sensitivity remained unchanged, reinforcing CDADC1’s specificity for 2′-deoxynucleotide analogs (*SI Appendix*, Fig. S5*D*).

We conclude that endogenous CDADC1 contributes significantly to resistance to gemcitabine and decitabine.

### CDADC1 Deletion Sensitizes Epithelial Cancer Cells to Gemcitabine In Vitro and In Vivo.

**Fig. 4.**
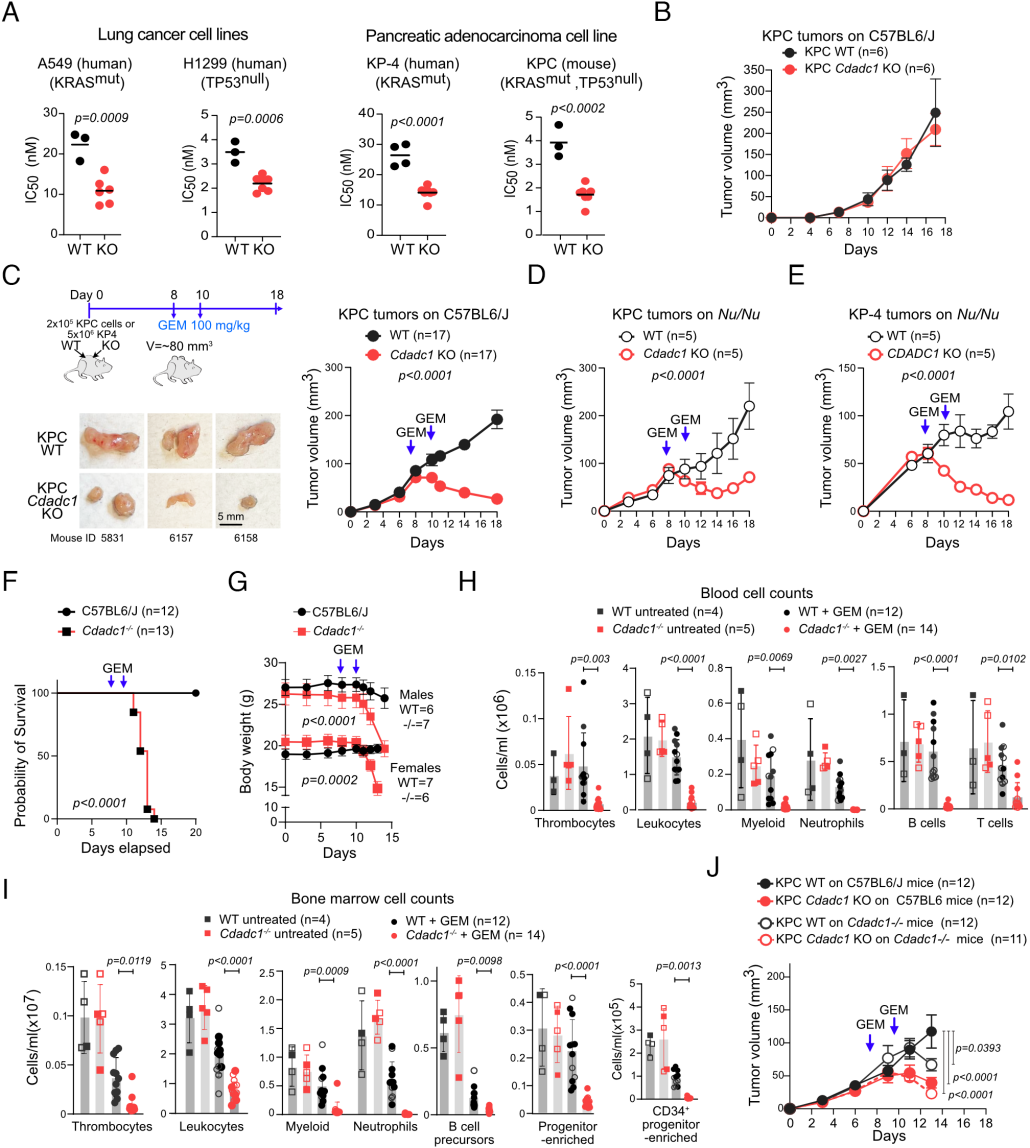
CDADC1 deletion sensitizes epithelial cancer cells to gemcitabine. (*A*) Mean (lines) and individual IC50 values for biological replicates (symbols) of isogenic WT and CDADC1 KO cell lines. Data from three independent experiments (2 for KP-4). P-values from the unpaired two-tailed *t* test. (*B*) Growth of ectopic KPC WT and *Cdadc1* KO tumors in untreated C57BL/6 J mice, with each mouse bearing both tumor types. Mean ± SEM of six tumors per genotype (*C*) Experimental design for tumor treatment and KPC WT and *Cdadc1* KO tumor size over time. Mean ± SEM from 17 mice across four experiments. *P*-value from two-way ANOVA. Representative images of KPC tumors from day 18. (*D*) Growth of KPC tumors, as in (*C*) but on athymic mice (*Nu/Nu*). (*E*) Growth of KP-4 cells tumors on athymic mice, analyzed as in (*C*) (*F*) Kaplan–Meier survival plot of WT and *Cdadc1^−/−^* littermate mice bearing KPC tumors and treated with gemcitabine (50 mg/kg), as in (*C*) from two independent experiments. *P*-value by long-rank test. (*G*) Mean ± SEM weight over time for mice in (*F*), plotting males and females separately. *P*-values from the two-way ANOVA test. (*H*) Concentration of blood leukocyte populations in mice treated as in (*F*). Paired WT mice were killed when *Cdadc1^−/−^* reached endpoints ~ day 3 to 4 after the second gemcitabine dose. *P*-values from one-way ANOVA with Tukey’s multiple comparison. For clarity, only comparisons between treated mice are shown. Differences between treated *Cdadc1^−/−^* mice and untreated mice were also significant. No significant differences between untreated mice. (*I*) Bone marrow leukocyte populations, plotted and analyzed as in (*H*). (*J*) KPC WT and *Cdadc1* KO tumor size over time in C57BL6/J WT or *Cdadc1^−/−^* mice from (*F*). *P*-values from two-way ANOVA with Bonferroni’s posttest.

Gemcitabine is the standard treatment for pancreatic, lung, and other epithelial cancers. We generated CDADC1-deficient variants of A-549, H1299, and KP-4 human cell lines, representing lung adenocarcinoma, highly proliferative p53-deficient large-cell lung carcinoma, and aggressive pancreatic ductal adenocarcinoma, respectively. *CDADC1* inactivation significantly increased gemcitabine sensitivity in all cell lines (Fig. 4*A* and *SI Appendix*, Fig. S5*E*).

To test the relevance of CDADC1 in gemcitabine resistance in vivo, we used the KPC1245 (KPC) pancreatic cancer cell line, derived from *Kras Trp53* mutant mice, which can form ectopic tumors and is used for preclinical studies (37). *Cdadc1* inactivation significantly sensitized KPC cells to gemcitabine (Fig. 4*A* and *SI Appendix*, Fig. S5*E*). *Cdadc1* KO and WT KPC cells grew equally in vitro (*SI Appendix*, Fig. S6*A*) and formed tumors of comparable size when implanted into C57BL6/J mice (Fig. 4*B*). Thus, CDADC1 deficiency did not intrinsically affect KPC cell growth or elicited rejection in vivo in the absence of any treatment.

We repeated this procedure but administered gemcitabine once tumors reached ~80 mm^3^ (Fig. 4*C*). While WT tumors showed minimal response to the treatment, *Cdadc1* KO tumors regressed significantly (Fig. 4*C*). Histological analysis showed fewer mitotic figures in treated *Cdadc1* KO than WT tumors, but similar average number of apoptotic cells at endpoint (*SI Appendix*, Fig. S6 *B* and *C*), albeit by this time tumors greatly differ in size. Indeed, in vitro assays showed that *Cdadc1* KO KPC cells underwent more apoptosis than WT upon gemcitabine treatment (*SI Appendix*, Fig. S6*D*).

CD3^+^ T cells were present around both KPC WT and KO tumors, with significantly more infiltrating KO tumors (*SI Appendix*, Fig. S6*E*). This may reflect improved access due to reduced stroma in smaller KO tumors and/or an enhanced immune response. To test this, we repeated the experiment in athymic (T-cell deficient) mice. Despite a slightly weaker response than in C57BL/6 J mice, *Cdadc1* KO KPC tumors remained substantially more sensitive to gemcitabine than WT tumors (Fig. 4*D*). Similarly, *CDADC1* KO human KP-4 pancreatic cancer cells showed strong gemcitabine sensitivity in athymic mice (Fig. 4*E*).

Thus, CDADC1 promotes gemcitabine resistance in human cancer cells, and its deletion enhances gemcitabine efficacy in vivo, primarily by increasing cytostatic and apoptotic effects, although we cannot formally rule out a contribution of the adaptive immunity.

### High Gemcitabine Toxicity in CDADC1-Deficient Mice.

We found that *Cdadc1^−/−^* mice treated with gemcitabine displayed weight loss (≥20%) and diarrhea, rapidly reaching humane endpoints for euthanasia as defined by Institut de Recherches Cliniques de Montreal (IRCM) animal use protocol 2025-1302 / 2024-09 JDN (Fig. 4 *F* and *G*). Gemcitabine caused pronounced thrombocytopenia and leukopenia, with highly reduced myeloid and lymphoid cells within the bone marrow, in *Cdadc1^−/−^* mice (Fig. 4 *H* and *I* and *SI Appendix*, Fig. S7 *A* and *B*). The strong decrease in B cell development stages and other bone marrow populations enriched in hematopoietic precursors, but not in the quiescent recirculating mature B and T cells, indicated that gemcitabine was particularly toxic to Cdadc1-deficient dividing cells (Fig. 4*I* and *SI Appendix*, Fig. S7*C*), despite *Cdadc1* is similarly expressed in all immune cell types whether replicative or quiescent (*SI Appendix*, Fig. S7*D*).

Despite the systemic effects, *Cdadc1* KO KPC tumors continued to respond more effectively to gemcitabine than WT tumors in *Cdadc1^−/−^* mice (Fig. 4*J*). Additionally, WT tumors in *Cdadc1^−/−^* mice seemed to respond better than those in WT mice, suggesting that systemic Cdadc1 influenced gemcitabine availability (Fig. 4*J*).

We conclude that Cdadc1 has an essential role in metabolizing gemcitabine in mice, thereby mitigating toxicity in proliferating cells.

### Mechanism of dC Analog Inactivation by CDADC1.

**Fig. 5.**
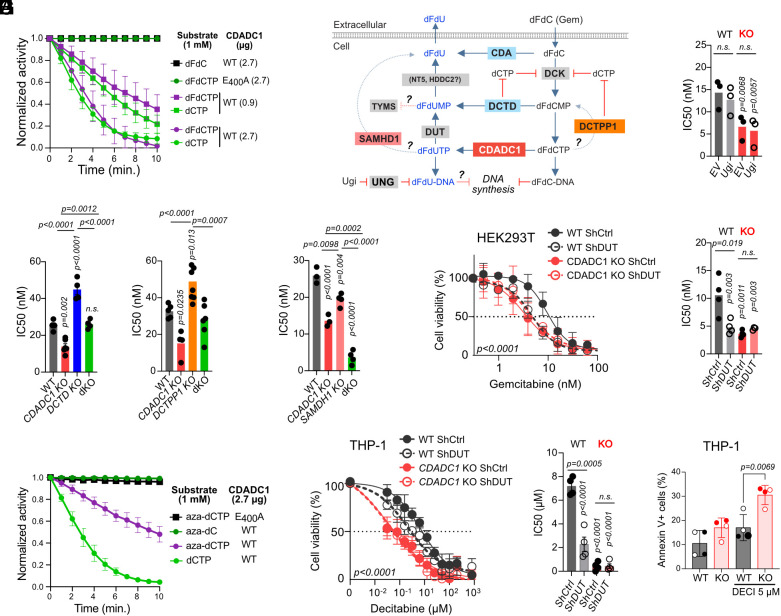
Mechanisms of CDADC1-mediated gemcitabine resistance. (*A*) Activity of recombinant CDADC1 on dCTP and gemcitabine nucleoside (dFdC) or dFdCTP monitored by absorbance at 290 nm. Means ± SEM from three reactions with independent protein preparations. (*B*) Schematic pathway of cellular dFdC metabolism. Question marks indicate reactions with limited evidence. (*C*) IC_50_ for gemcitabine of HEK293T WT and *CDADC1* KO cells expressing lentiviral empty vector (EV) or encoding the UNG inhibitor Ugi, grown in the presence of gemcitabine for 72 h. Vertical *P*-values from one-way ANOVA with Tukey’s posttest (versus WT EV). (*D* and *E*) IC_50_ for gemcitabine of HEK293T cells of the indicated genotypes. *P*-values from one-way ANOVA with Tukey’s posttest. (*F*) Viability curves (mean ± SD from four experiments) and IC_50_ (mean and individual values for biological replicates) for gemcitabine of HEK293T WT and *CDADC1* KO cells expressing shRNAs targeting DUT (shDUT) or control (shCtrl). *P*-values from two-way (curves) or one-way (scatter plot) ANOVA with Tukey’s posttest. (*G*) Activity of recombinant CDADC1 on dCTP and decitabine nucleoside (aza-dC) or aza-dCTP monitored by absorbance at 230 nm. Mean ± SEM from three reactions with independent protein preparations. (*H*) plot and analysis as in (*F*) for THP-1 and decitabine. (*I*) Apoptosis in WT and CDADC1 KO THP-1 cells treated with decitabine for 72 h. Mean (bars) and individual values for biological replicates from four experiments with two clones each (different symbols). (*C*, *F*, *H*, and *I*) Horizontal *P*-values from the unpaired two-tailed *t* test.

CDADC1 robustly deaminated dFdCTP, albeit slightly less efficiently than dCTP (Fig. 5*A*). In contrast, it showed no activity on the dFdC nucleoside (Fig. 5*A*) and only weakly deaminated cytarabine triphosphate, comparable to its activity on CTP (*SI Appendix*, Fig. S8*A*).

CDADC1-catalyzed dFdUTP could be rendered inactive by several pathways, such as base excision by UNG or hydrolysis by nucleotidases like SAMHD1 (38) and DUT (39) (Fig. 5*B*). Alternatively, these or other enzymes (e.g., DCTPP1 that may compete for dFdCTP) (Fig. 5*B*) could function independently of CDADC1 and reveal cooperative effects. To test these options, we generated HEK293T with deficiencies in each of these enzymes, alone or in combination with *CDADC1* KO, and compared their relative sensitivity to gemcitabine. UNG inhibition by expressing Ugi (a phage-derived specific inhibitor), had no effect in either condition (Fig. 5*C* and *SI Appendix*, Fig. S8*B*). Inactivating *DCTD* or *DCTPP1*, enzymes that reduce cellular dCTP levels, significantly increased gemcitabine resistance, but CDADC1 deletion restored sensitivity to WT levels (Fig. 5*D* and *SI Appendix*, Fig. S8 *C* and *D*), suggesting competition. On the other hand, *SAMHD1* inactivation sensitized cells to gemcitabine and showed cooperativity with *CDADC1* KO (Fig. 5*E* and *SI Appendix*, Fig. S8*E*), indicating they acted independently. Finally, DUT depletion sensitized WT cells to gemcitabine but did not further sensitize *CDADC1* KO cells, both in HEK293T or THP-1 cells (Fig. 5*F* and *SI Appendix*, Fig. S8 *F* and *G*), indicating DUT was epistatic downstream from CDADC1.

We confirmed that the basic mechanism of inactivation was shared between gemcitabine and decitabine. CDADC1 deaminated decitabine triphosphate but not its nucleoside, albeit less efficiently than dCTP (Fig. 5*G*). This is likely due to the substitution of a nitrogen for the carbon in position 5 of the pyrimidine ring of decitabine. CDADC1 and DUT were also epistatic for decitabine toxicity (Fig. 5*H*). Finally, CDADC1 deficiency enhanced the apoptotic effect of decitabine (Fig. 5*I*).

We conclude that CDADC1 directly deaminates dFdCTP and 5-aza-dCTP, rendering the products susceptible to DUT, which prevents their genomic incorporation and apoptotic effects.

## Discussion

We report two major findings: the identification of CDADC1 as a vertebrate-specific dCTP deaminase and its critical role in reducing the toxicity of gemcitabine and decitabine in replicating cells. CDADC1 deficiency sensitizes cancer cells to these drugs, but full-body deficiency leads to toxicity in gemcitabine-treated mice.

The enzymatic deamination of dCTP to generate dUTP has been described in bacteria, viruses, and archaea but is unprecedented in eukaryotes (7, 9, 10, 40). Previous biochemical characterization of CDADC1 was performed using recombinant protein partially purified from *E. coli* (41). Their assignment of cytidine deaminase activity likely stemmed from contamination of recombinant CDADC1 preparations with *E. coli* cdd, which similarly confounded the initial characterization of AID and APOBECs (32). Our biochemical data, using highly purified CDADC1 and catalytically inactive control, show that CDADC1 specifically deaminates dCTP, excluding cytidine, deoxycytidine, and dCMP as substrates. CDADC1’s preference for dCTP is further supported by its activity on dFdCTP and 5-aza-dCTP over their nucleosides. We cannot formally exclude a function for the weak activity of CDADC1 on CTP, but CDADC1 overexpression affecting dCTP and not CTP pools, and CDADC1 selectively impacting sensitivity to dC analogs, suggest that dCTP is the preferred cellular substrate, with dUTP as product.

The presence of DUT and uracil excision repair in most species indicate that dUTP and uracil in DNA are generally detrimental (42, 43). However, uracil in DNA is functional during antibody diversification (44), and UNG-deficient mice and humans show that vertebrates can tolerate uracil in DNA with relatively minor phenotypic consequences (45–47). The identification of CDADC1’s biochemical activity now implies a physiological role for dUTP in vertebrates.

While the biological function of CDADC1 remains unknown, we can rule out that CDADC1 contributes significantly to dTTP biosynthesis for cell proliferation, in contrast to bacterial *dcd*. Indeed, endogenous CDADC1 does not alter dNTP pools, lacks allosteric regulation by dTTP, and is dispensable for proliferation in cell lines and mouse tissues. Further work is required to identify the biological function that drove the evolution of CDADC1 in vertebrates. Some Gram-negative bacteria use Zn-dependent dCTP deaminases to deplete dCTP and hinder phage replication. These deaminases are fused to other activities and are not CDADC1 homologs (48, 49), but raise the possibility that CDADC1 restricts specific viruses.

Our data do establish CDADC1’s dCTP deaminase activity as a metabolic reaction influencing cellular sensitivity to gemcitabine and decitabine, with dual clinical implications.

On one hand, CDADC1 deficiency enhances the sensitivity of human cancer cells to gemcitabine and decitabine, increasing the antitumor efficacy of gemcitabine in mouse models of pancreatic cancer at a dose equivalent to ~380 mg/m^2^. The standard dose in human patients is ~1,000 mg/m^2^ (50). While CDA inactivates dC analog by converting them to dU nucleoside derivatives, which are poor DCK substrates (23), CDADC1 uses a different mechanism. CDADC1 deaminates dFdCTP and 5-aza-dCTP, generating dFdUTP and 5-aza-dUTP, respectively, which are hydrolyzed by DUT to their monophosphate forms. DCTD also generates dFdUMP and 5-aza-dUMP. DCTD was initially thought to counter dC analog toxicity, but it has since been shown to enhance it (21, 22), presumably because the monophosphate drugs are competitive inhibitors of TYMS in vitro (51). How can DCTD and CDADC1 have opposing effects on gemcitabine toxicity if both produce the same monophosphate metabolites? We propose that DCTD deficiency increases dC analog resistance primarily by elevating cellular dCTP (34, 35, 52), which allosterically inhibits DCK, the rate-limiting enzyme in dC analog activation (53). Increased resistance of DCTPP1-deficient cells to gemcitabine (39, 54), could be similarly explained. However, we note that the interplay between CDADC1 and DCTTP1 may differ for gemcitabine and decitabine. Indeed, *DCTPP1* KO HeLa cells are more sensitive to decitabine (39). Future studies will clarify these differences. Nonetheless, our data establish a common minimal pathway for both drugs, in which CDADC1 directly deaminates gemcitabine and decitabine triphosphates. This activity followed by hydrolysis via DUT activity prevents their incorporation.

On the other hand, *Cdadc1^−/−^* mice are highly sensitive to gemcitabine, with symptoms resembling those described mice and humans displaying low CDA activity (23, 55, 56). Gemcitabine toxicity in *Cdadc1^−/−^* mice likely reflects depletion of rapidly dividing cells, as suggested by diarrhea and loss of hematopoietic precursors in bone marrow in all treated mice. This level of toxicity was unexpected, given that CDA is considered the primary detoxifying enzyme for gemcitabine (23). Since CDADC1 is insensitive to THU, our findings indicate that CDADC1 and CDA play nonredundant detoxifying roles. The clinical implications of these findings are two-fold.

First, CDADC1 inhibition could enhance dC analog efficacy. A priori, this approach would have a narrow therapeutic index because of toxicity. However, pharmacological inhibition may be less drastic than genetic deficiency, or an inhibitor could be selectively delivered to cancer cells [e.g., via lipid nanoparticles (57)]. Synthetic lethality strategies may also mitigate toxicity by enabling lower inhibitor doses. For example, CDADC1 inhibition may synergize with SAMHD1 inhibitors, as suggested by our results. *CDADC1* expression was largely similar in cancer versus corresponding normal tissues but appeared higher in pancreatic cancer (*SI Appendix*, Fig. S9*A*), where gemcitabine is standard of care. CDADC1 expression was not prognostic in pancreatic or most other cancers, except for kidney carcinoma and lung adenocarcinoma in which higher levels correlated with longer survival (*SI Appendix*, Table S1). This association may reflect reduced cellular fitness due to DNA damage caused by dUTP incorporation, consistent with CDADC1 overexpression reducing growth in cell lines (41, 58). A fraction of bladder, sarcoma, and prostate cancer samples showed very low CDADC1 expression, correlating with *CDADC1* copy number losses (*SI Appendix*, Figs. S5*B* and S9*B*). This loss may be due to its proximity to the *RB1* tumor suppressor locus in chromosome 13 (59). Cancers with low CDADC1 may be naturally more sensitive to dC analogs or differentially benefit from CDADC1 inhibition while avoiding severe toxicity.

Second, should CDADC1 be as critical in humans as is in mice, patients with low CDADC1 or loss-of-function mutations would be at high risk of therapy-associated toxicity when administered gemcitabine, and perhaps also decitabine. CDA, currently considered the main detoxifying enzyme for dC analogs, has well-characterized clinical variants with reduced activity, but genotype-to-phenotype relationships regarding toxicity remain inconsistent (23, 24). CDADC1 could underlie toxicities unexplained by CDA defects. The NCBI ClinVar database lists 22 CDADC1 coding variants of unknown significance, and gnomAD reports 410 missense mutations in *CDADC1* that warrant characterization.

In conclusion, our findings suggest that current models of dC analog metabolism should be revised to incorporate CDADC1 when evaluating clinical treatment suitability, for a full understanding of drug pharmacodynamics and toxicity.

## Materials and Methods

Detailed methods are available as *SI Appendix*.

### Monitoring Deaminase Activity.

Human CDADC1 and DCTD were purified from *E. coli* dcd by His-Select nickel affinity chromatography, dialyzed, and stored at −80 °C. Enzymatic reactions were performed in 50 mM Tris-HCl, pH 7.5, with 1 to 2 mM substrate, monitoring deamination by decrease in absorbance at 290 nm or by thin layer chromatography versus appropriate standards.

### Gene Targeting in Cell Lines.

Mouse (*Cdadc1* and *Dctd*) and human (*CDADC1*, *DCTD*, *DCTTP1*, and *SAMHD1*) genes were inactivated using CRISPR/Cas9. Cell growth was assessed via crystal violet staining, CCK-8 assay, or viable cell counting. Apoptosis was measured with Annexin V.

### Cdadc1-Deficient Mice.

*Cdadc1^−/−^* mice were generated by CRISPR/Cas9 targeting exon 4 and backcrossed to C57BL6/J for >10 generations. Lifespan and fertility were monitored against matched littermates obtained from *Cdadc1^±^* crosses.

### Ectopic Pancreatic Cancer Mouse Models.

Cel lines were injected subcutaneously into C56BL6/J, *Cdadc1^−/−^*, or athymic (nu/nu) mice. Gemcitabine (50 to 100 mg/kg, i.p.) was administered at 60 to 100 mm^3^ tumor size and again 48 h later. Tumor growth was monitored, and mice were euthanized when the tumor reached a volume of 1 cm³, or when advised by veterinary inspection due to weight loss ≥20%, lethargy, dehydration, or tumor ulceration according to IRCM animal use protocol 2025-1302 / 2024-09 JDN. All procedures were approved by the IRCM Animal Protection Committee following Canadian Council on Animal Care guidelines.

## Supplementary Material

Appendix 01 (PDF)

Dataset S01 (XLSX)

## Data Availability

All study data are included in the article and/or supporting information.
