# Supplementary material for: CDADC1 is a vertebrate-specific dCTP deaminase that metabolizes gemcitabine and decitabine to prevent cellular toxicity
Source: Proc Natl Acad Sci U S A. 2025 Jun 12;122(24):e2424409122. doi: 10.1073/pnas.2424409122 (PMC12184417; doi:10.1073/pnas.2424409122)
Supplement: Supplementary file 1 — Appendix 01 (PDF) [file pnas.2424409122.sapp.pdf]

PNAS

Supporting Information for

CDADC1 is a vertebrate-specific dCTP deaminase that metabolizes gemcitabine and decitabine to prevent cellular toxicity

Marcelo M. Rodriguez[1,9], Debashree Chatterjee[1,9], Johanna Guerry[1,2], Anne-Marie Patenaude[1], Charles C.H. Cohen[1,2,], Therence Bois[1,2], Ariane Larouche[1], Silvana Ferreira[1], Thierry Bertomeu[3,4], Andrew Chatr-aryamontri[3,4], Li Zhang[3,4], Sylvie Mader[4], Corey Nislow[5], Guillaume St-Jean[6], Yvan Guindon[1,7], Astrid Zahn[1], Javier M. Di Noia[1,2,8,*].

[1] Institut de Recherches Cliniques de Montréal, Montréal, Québec, H2W 1R7, Canada.
[2] Molecular biology programs, Université de Montréal, Montréal, Québec, H3C 3J7, Canada.
[3] Chemogenix, Université de Montréal, Montréal, Québec, H3T 1J4, Canada.
[4] Institute for Research in Immunology and Cancer (IRIC), Université de Montréal, Montréal, Québec, H3T 1J4, Canada.
[5] Faculty of Pharmaceutical Science, University of British Columbia, Vancouver, British Columbia V6T 1Z3, Canada.
[6] Department de Pathology and Microbiology, Faculty of Veterinary Medicine, Université de Montréal, Saint-Hyacinthe, Québec, J2S 2M2, Canada.
[7] Department of Chemistry, Université de Montréal, Montréal, Québec, H3C 3J7, Canada.
[8] Department of Medicine, Université de Montréal, Montréal, Québec, H3C 3J7, Canada.
[9] Equal contributions.

*Corresponding author Javier M Di Noia, Email: javier.di.noia@ircm.qc.ca

This PDF file includes:

Supporting text
Figures S1 to S9
Tables S1 to S3
Legends for Datasets S1
SI References

Other supporting materials for this manuscript include the following:

Dataset S1

DETAILED MATERIALS AND METHODS

Molecular cloning.

The open reading frame of human CDADC1 isoform 1 (v1) (NP_112173.1) was amplified from Ramos B cells by RT-PCR using ProtoScriptTM M-MuLV Taq RT-PCR kit (NE BioLabs) and oligonucleotides designed on sequences obtained from GenBank EST pileups and predicted transcripts from ENSEMBL. Amplicons were cloned in pGEM-T Easy (Promega) and sequenced. Human DCTD (XP_005262835) was similarly cloned. *E. coli* codon-optimized human CDADC1v1 open reading frame was synthesized at Integrated DNA Technologies Inc (Coralville, IO, USA). A catalytically inactive variant ($E_{400}A$) was produced by site directed mutagenesis using KOD Hot Start DNA Polymerase (Sigma, cat# 71086) with oligonucleotides OJ1125/OJ1126 (original sequence) or OJ3591/OJ3592 (*E. coli* codon optimized version). Bacterial expression vectors were generated by subcloning into pTrcHis-C or pTrcHis-A (ThermoFisher). Lentiviral vectors for doxycycline-inducible expression of human CDADC1v1 and $E_{400}A$ mutant were constructed by cloning into pEN_tmcs at NotI/SacII (Addgene plasmid # 25751) and transferred by Gateway cloning into pSLIK-Neo (1) (Addgene plasmid # 25735). Two complementary oligonucleotides (OJ2849/OJ2850) encoding an antisense RNA targeting human DUT were annealed and ligated into the lentiviral vector pLKO.1 neo (Addgene #13425) using AgeI and EcoRI sites. The coding region of the bacteriophage-derived Ugi protein was amplified with primers OJ1902/OJ1903 from pEF-Ugi (2) and cloned into the PacI site of lentiviral vector pWPI (Addgene #12254). All oligonucleotide sequences are provided in **Table S2**.

Recombinant protein purification.

Human CDADC1 and DCTD cloned into pTrcHis were transformed into in *E. coli* BW1105 (KL16 dcd-12::kan), a reconstruction of BW1040 (3) kindly provided by Dr. Bernard Weiss (Emory University, GA). Typically, 5 ml starter cultures from a single colony of BW1105 transformed with either pTrcHis-CDADC1v1 or pTrcHis-DCTD were grown overnight in LB or 2XTY medium at 37°C supplemented with ampicillin (100 µg/ml), kanamycin (35 µg/ml) and thymidine (150 µg/ml), and used to inoculate 300-500 ml of the same media to reach a starting OD_{600} ~0.03, which was grown at 37°C to an OD_{600} ~0.6 before adding IPTG to a final concentration of 0.5 mM and culturing overnight at 18°C. Cells were harvested by centrifugation in a Beckman Coulter centrifuge in JA-10 rotor at 3,500 x g for 20 min at 4°C. All subsequent steps were conducted at 4°C. The cell pellet was suspended in 13 ml of equilibration buffer [50 mM sodium phosphate pH 8, 300 mM NaCl, 1 mM dithiothreitol (DTT), 10 µM ZnSO4, 10 mM Imidazole, 1.5 mM PMSF and 0.25X complete EDTA-free protease inhibitor (Roche)]. Bacteria were disrupted on ice by sonication using a Branson sonic disruptor (ThermoFisher) 8 cycles each consisting of 10 seconds pulse ON with 30 sec OFF intervals at 70% amplitude. The lysate was clarified by centrifugation at 10 000 rpm 20 min. The supernatant was passed through a 0.45 µm filter and adjusted to 1% Triton X-100. His-Select nickel affinity gel (1 ml of 50% slurry) (Sigma Aldrich) was added and gently mixed for 1 h at 4°C. The resin-extract mix was loaded in a poly-prep column (Bio Rad) and washed 3 times with 10 column volumes of washing buffer [50 mM sodium phosphate pH 8, 300 mM NaCl, 1 mM DTT, 10 µM $ZnSO_4$, 20 mM Imidazole]. The column was eluted in fractions of 0.5 or 1 ml with elution buffer [50 mM sodium phosphate pH 8, 300 mM NaCl, 1 mM DTT, 10 µM, $ZnSO_4$, 250 mM Imidazole]. Aliquots of 60 µl from each fraction were analyzed by SDS-PAGE. Typically, fractions 1-2 contained most recombinant protein and were pooled and dialyzed overnight in 50mM Tris-HCl, pH 7.5, 1 mM DTT, 10 µM $ZnSO_4$, 1.5 mM

PMSF, 1X complete protease inhibitor, 10% glycerol. Protein concentration was estimated from serial dilutions of the final preparation compared to BSA standards in Coomassie blue-stained SDS-PAGE. Dialyzed preparations were aliquoted and stored at -80°C and thawed once for activity measurements.

Monitoring deaminase activity.

Enzymatic reactions were typically carried out in 50 mM Tris-HCl, pH 7.5, 1-2 mM substrate, in a final volume of 100 μl in UV transparent plates (Corning). Reaction mixtures equilibrated at 37°C were started by the addition of purified enzyme and incubated at 37°C for 10 min. Reactions were sampled every 20 seconds by measuring absorbance at 290 nm in a SpectraMax spectrophotometer (Molecular devices). Because of the difference in extinction coefficient, cytosine containing nucleosides and nucleotides absorb at 290 nm while uracil containing ones do not, thus we recorded decrease in absorbance nm (4, 5). To test CDADC1 substrate specificity we used 1 mM of CTP, dCMP, dC, cytidine, 5'-methyl-dCTP in reactions containing 0.9 ug or 2.7 ug CDADC1. Allosteric modulators. For DCTD 0.1 mM dCTP and 1 mM dTTP were added to reactions containing 30-50 ng DCTD, 1 mM dCMP substrate, where indicated. To test possible activating or inhibitory effects on the CDADC1 dCTP deaminase activity 0.5 mM of dCTP or dTTP were added in reactions with 50 ng CDADC1, 1 mM dCTP substrate. Deamination of gemcitabine-5'-triphosphate and cytarabine-5'-triphoshate (Jena Bioscience GmbH) were monitored identically. Deamination of decitabine-5'-triphosphate (Jena Bioscience GmbH) was monitored at 230nm, based on its maximum absorbance of 221 nm (6). Results were expressed as absorbance change compared to the initial time, subtracting the background absorbance of the corresponding uracil nucleotide. All the reactions were performed at least twice with independent enzyme preparations. Where indicated, 10 or 25 nM tetrahydrouridine (THU) (MedChemExpress # HY-15345A) was pre-incubated for 1 min with CDADC1 before adding dCTP substrate and the reaction was immediately monitored as above. Control reactions with human 1 μg CDA (ProSpec #ENZ-007) and 1 mM dC substrate were performed in parallel to confirm THU activity.

Thin-layer chromatography

For product identification, reactions were incubated 15 min as above and substrate and products separated by thin-layer chromatography. Aliquots of 10-15 μl from the enzymatic reaction mixtures were applied to PEI-cellulose TLC plates (Sigma Aldrich) and dried in a vacuum chamber. To separate dCMP from dUMP, dCTP from dUTP or CTP from UTP, the plates were run with ascending capillarity in a saturated chamber, stepwise by solutions a) 1 M acetic acid for 1 min, b) 1 M acetic acid 0.3 M LiCl to 1.5 or 3 cm above the starting line (for a 7 or 16 cm long paper, respectively), c) 3M acetic acid 2M LiCl to ~2 cm below the top of the paper (7). For separating (deoxy)cytidine from (deoxy)uridine the plates were run continuously in isobutyric acid : water : 25% NH_4OH (100 ml : 52 ml : 0.1ml) (4). The plates were washed in 100% methanol for 5 min and dried with hot air, and the nucleotide spots were a.

Enzyme kinetics.

CDADC1 and DCTD enzymatic mixtures were equilibrated at 37 °C for 15 min. Reactions were started by the addition of 0.1 - 4 mM substrate and monitored at 290 nm every 20 s. Initial velocity of the reaction was calculated by linear regression as the slope of the linear portion of the

absorbance versus time curve. This value (A) was transformed to concentration of product produced per minute from the Beer-Lambert law equation (A = e *C L*) using e = 1.69 x 10^3 M^{-1} cm^{-1} (the difference in extinction coefficient at 290 nm between deoxycytidine and deoxyuridine at pH 7.5) (8) and light path L = 0.29 cm for the 96 well plates used. These values were converted to µmole of product generated per min per mg of enzyme to calculate the initial enzymatic velocities (V_0). The V_0 plotted as a function of substrate concentration were fitted by nonlinear regression to either Michaelis-Menten or sigmoidal curve. Maximal velocity (Vmax), Menten constant (Km), and Hill coefficient (h) were determined from the fitted curve using Prism (GraphPad).

E. coli mutation assay to monitor DNA deamination.

E. coli strain BW310 (KL16 *ung-1*) was transformed with AID or CDADC1 subcloned into pTrcHisA (Invitrogen). Isolated colonies were picked into 2XTY media 100 µg/ml carbenicillin and grown overnight. Cells were diluted 1:50 in the same medium and grown for ~6 h (until OD_{600} ~0.5) before adding Isopropyl β-D-1-thiogalactopyranoside (IPTG) to 1 mM final concentration and culturing for another 3 h. Appropriate dilutions were then plated on LB agar plates with either ampicillin (100 µg/ml) or rifampicin (100 µg/ml). Mutation frequency in each individual culture was calculated as the median number of colonies that survived rifampicin selection per 10^9 ampicillin-resistant (viable) cells and median values and distributions compared.

Bacterial complementation assays.

E. coli strain BW1105 (KL16 dcd-12::kan) was grown in LB medium supplemented with 125 µg/ml thymidine (Sigma) to prevent reversion and made competent for transformation. Transformed bacteria were plated in LB plates with antibiotic selection and 125 µg/ml thymidine. Individual colonies were grown in 2XYT + 125 µg/ml thymidine for 6 h, resuspended in M9 medium, diluted to OD_{600} = 1 and plated on minimal medium (1% m/v $MgSO_4$, 10% Citric acid, 50% K_2HPO_4 phosphate, 17% $NaNH_4HPO_4$) plates with or without IPTG and/or 125 µg/ml thymidine and grown at 37C for 72 h.

Cell lines and culture.

MOLT-4, K562 (ATCC obtained through Dr. Tarik Moroy, IRCM), and H1299 (ATCC obtained through Dr. KelsieThu, University of Toronto) cells, were cultured in RPMI 1640 (Wisent) supplemented with 10% FBS (Wisent). Jurkat cells (subclone E6, a kind gift of Dr. E Cohen, IRCM) were cultured in RPMI 1640 (Wisent) 10% heat inactivated FBS (Wisent), 1 mM L-glutamine (Wisent). CH12F3 B cells (9) (a gift from Dr. T Honjo, Kyoto University) and THP-1 (ATCC, obtained through Dr. Eric Cohen) were cultured in RPMI 1640 (Wisent) supplemented with 10% FBS (Wisent) and 100 mM b-mercaptoethanol (Wisent). A549 cells (ATCC obtained through Dr. Kelsie Thu, University of Toronto) were cultured in DMEM/F12 (Wisent) supplemented with 10% FBS (Wisent). KP-4 (obtained from Dr. Gerardo Ferbeyre, Université de Montréal) and HEK293T cells (ATCC) were cultured in DMEM 10% FBS with10 mM HEPES. KPC1245 cells originally from Dr. David Tuveson's (Cold Spring Harbor laboratory) were obtained from Dr. John Stagg (University of Montreal) and were cultured in DMEM (Wisent) supplemented with 10% FBS (Wisent). All media were supplemented with penicillin streptomycin (Wisent). Cells were cultured at 37˚C in humidified 5% CO_2 incubators. Cells were regularly checked for mycoplasma and all experiments were performed with mycoplasma-free cultures. Cell clones with DCTD KO (either single or in combination with CDADC1 KO) were expanded and maintained in

media supplemented with 50 mM dNTP mix (NEB # N0447L, or BioBasic # DD0056). Nucleotide supplementation was removed for experiments.

Gene inactivation by CRISPR/Cas9.

Guide RNA (gRNA) sequences were designed using CRISPR Design Tool (http://tools.genome-engineering.org) and synthesized as two complementary oligonucleotides (All oligonucleotide sequences in **Table S2**). Two gRNAs designed to produce a small genomic deletion that facilitated screening were simultaneously used in every case. For mouse *Cdadc1*, we targeted exon 4 (OJ1234/5 and OJ1236/7) and for *Dctd*, we targeted exon 1 and exon 2 (OJ1163/4 and OJ1165/6*).* For human *CDADC1*, we designed gRNAs targeting exon 1 (OJ928/9 and 930/1), exon 4 (OJ936/7 and OJ938/9), and exon 5 (OJ932/3 and OJ934/5); and for *DCTD*, we targeted exons 2 and 3 (OJ1147/8 and OJ1149/50). For human *DCTPP1*, we designed gRNAs targeting exon 1 or exon 2 (OJ3420/1 and OJ3422/3, respectively), and for *SAMHD1* exon 2 (OJ3598/9). The annealed oligonucleotides encoding each gRNA were ligated into plasmids pX458 (pSpCas9(BB)-2A-GFP, Addgene #48138), pX459 (pSpCas9(BB)-2A-Puro, Addgene #48139), or LentiCRISPRv2-mCherry (Addgene #99154) lentiviral vector. Most cell lines were co-transfected with both pX458-gRNA vectors. MOLT-4, THP-1 and JURKAT cells were transduced by spin-infection with LentiCRISPRv2-mCherry lentiviruses as described below. CH12F3 was transfected by electroporation using nucleofector kit V (Amaxa), following the manufacturer's protocol. HEK293T, KPC, A549 and H1299 cells were transfected by TransIT-LT1 Reagent (Mirus) and K562 and KP4 cells were transfected by Lipofectamine 2000 **(**Thermofisher # 11668019). After transfection with pX458 (GFP) vectors, we sorted single GFP+ cells into 96-well plates 48h post-transfection. MOLT-4 cells expressing LentiCRISPRv2-mCherry single mCherry+ cells were sorted in 96-well plates 48-72h after transduction. HEK293T cells transfected with pX459 (Puro) vectors were cloned by limiting dilution after puromycin selection. DNA from the resulting clones was extracted with Direct-PCR (Viagen) with Proteinase K (Biobasic) following the manufacturer's protocol. Cellular clones with the intended deletion were identified by PCR as follows: *Cdadc1*, oligonucleotides OJ1128 and OJ1229 4; *Dctd*, OJ1157-OJ1158; *CDADC1*, OJ1028/OJ1029 or OJ2324/2325 for exon 4, OJ1026/OJ1057 for exon 1, OJ1030/OJ1031 for exon 5 strategies (exon 4 strategy was the most efficient in HEK293T and was used for all other cell lines), *DCTD*, OJ1151/OJ1152 (short product), or OJ2323/OJ1152 (long product)) or by the T7 Endonuclease I (NEB # M0302S) assay when a single gRNA was used. PCR products were Sanger sequenced either directly or after cloning in pGEM-T-Easy to identify deletions, frameshifts and premature stop codons. Supplementary **Table S3** summarizes information about all cell clones used in this work.

Lentiviral infections.

HEK293T cells were seeded at 0.5 x 10^6 cells per well in 2 ml media in 6-well plates and let to adhere overnight. Prior to transfection, media was changed for the media corresponding to the cells to be transduced with 10 mM HEPES. HEK293T cells were co-transfected with 2.6 µg of lentiviral vectors (LentiCRISPRv2-mCherry, pWPI, or pLKO.1 neo), 0.93 µg packaging vector psPAX2 (Addgene #12260) and 0.32 µg VSV-G envelope expressing vector MD2.G (Addgene #12259) (ratio 2:1:0.63) using *Trans*IT-LT1 Reagent (Mirus). Viral supernatant was collected 48 h post-transfection, filtered on 0.45 µm and frozen at -80ºC or used immediately for transduction. Supernatants with viruses encoding each of the two gRNAs to target the same gene were pooled. The target cells were plated at 0.4 x 10^6 cells, in 0.5 ml media in 24-well plate wells and

combined with 1.5-2 ml of viral supernatant adjusted to reach 8 µg/ml polybrene final after adding to cells. Plates were spun at 1600 rpm for 90 min at 30ºC; then incubated for 4h at 37 ºC. The medium was changed for 2 ml culture medium and cells incubated for 48 to 72 hours before sorting single cells or infected populations.

Monitoring cell growth and toxicity

Growth curves. Viable non-adherent cells were measured by Cell Counting Kit 8 (WST-8/CCK8) (Abcam #ab228554) or by manual counting. Cells were seeded at 1-5 x 10^4 cells in 24-well plates. At various times, cells were suspended by pipetting and 50 µl of cell suspension was taken and mixed with 50 µl of Trypan Blue (0,4% solution in PBS) (Multicell) and counted using a manual hemocytometer or a Countess 3 FL (Invitrogen). Adherent cells were seeded at 5 x 10^4 in 24-well plates and individual wells fixed with crystal violet as above at different times. The first time point was taken 4 h after seeding (day 0) and used to normalize the curves. Cell growth under stress conditions was tested by either growing cell lines in their normal medium but supplemented with 1% FBS (starvation) or transferring plates into a chamber filled with 5% O_2, 5% CO_2, 90% N_2, and incubated at 37C for 48 h (hypoxia). Cell lines CH12F3, HEK293T and THP-1 were plated at different densities (3 x 10^3 to 1 x 10^6 cells/mL for CH12F3; 2,2 x 10^5 to 7 x 10^5 cells/mL for HEK293T and 9 x 10^3 to 3 x 10^5 cells/mL for THP-1) in 24-well plates and cultured for 48 h before determining cell viability as above.

Cell survival for adherent cells lines. HEK293T, KPC, KP-4 A549, and H1299 cells were dispensed at 5 x 10^4 cells per well in a 24-well plate at 37˚C with 5% CO_2 for 24 h. Cells were treated once with 12 different concentrations of Cytarabine (Sigma, #C1768)) [0-10 mM] in PBS, or gemcitabine (MedChemExpress, #122111-03-9) [0-4000 nM] in PBS. For Decitabine (MedChemExpress, #2353-33-5) cells were treated daily with freshly prepared solution [0-100 µM] in DMSO. After 72 h, the cells were carefully washed 3 x with 1X PBS and fixed with cold methanol for 10 min. The methanol was then removed and cells incubated with crystal violet (0.2% in 20% Methanol) stain (Sigma) for 20 min. The plates were washed with ddH_2O three times and allowed to dry. 250 µl of acetic acid was used to dissolve the crystal violet stain for 5 min before adding 750 µl of ddH_2O. A 150 µl aliquot was transferred into 96-well plates and absorbance at 590 nm determined in a spectrophotometer SpectraMax® (Molecular devices).

Cell survival for non-adherent cell lines. Molt-4, CH12F3, Jurkat, K562 and THP-1 cell lines were dispensed at 1-5 x10^4 cells per well in a 24-well plate at 37˚C with 5% CO_2. Cells were treated as above. After 72 h of treatment (120 h for K562 cells), 100 µl of cell suspension were transferred into a 96-well plate and incubated with 10 µl of Cell Counting Kit 8. Absorbance at 460 nm was measured after incubating 2-3 h. For all cell viability assays, results were normalized to wells without the drug treatment (=100% viability).

Apoptosis by flow cytometry was measure using propidium iodide (Bioshop, #PPI888.10) and Annexin V APC (BD Pharmingen, # 550474) diluted in Annexin V Binding Buffer (BD Pharmingen, #556454).

Uracil-excision assay

Assays were performed as described (2). Briefly, total cell extracts of HEK293T cells were made by sonicating 3-4 x10^6 cells in 400 µL of HEN buffer [25 mM Hepes, 5 mM EDTA, 5 mM NaCl, 5 mM dithiothreitol, 10% glycerol, pH 7.5] with 1x Complete protease inhibitors (Roche #11697498001). The 42-mer reaction substrate was prepared by annealing OJ96 and OJ97, in

which OJ97 has a single centrally located uracil. Reactions containing 20 µg of cell extract (or dilutions thereof) and 1 pmol of substrate in 10 µl final volume in HEN buffer were incubated for 2 h at 37˚C. Reactions were stopped by adding 10 µl of formamide 1% phenol red loading buffer and boiling for 5 min. Substrate and product were resolved by 15% polyacrylamide TBE-Urea gel electrophoresis and imaged using a Typhoon™ FLA 7000 (GE Healthcare).

Measuring dNTPs by LC/MS

All LC/MS grade solvents and salts were purchased from Fisher (Ottawa, Ontario Canada): dichloromethane (DCM), water (H_2O), acetonitrile (ACN), methanol (MeOH) and ammonium acetate. The authentic standards for metabolites of interest were purchased from Sigma-Aldrich Co. (Oakville, Ontario, Canada). Cultured cells were washed with cold 150 mM ammonium formate solution (pH 7.4) and then extracted with 600 µL of 31.6% MeOH/36.3% ACN in H_2O (v/v). Cells were lysed and homogenized by bead-beating for 2 minutes at 30Hz using ceramic beads (TissueLyser II – Qiagen). Cellular extracts were partitioned into aqueous and organic layers following DCM treatment and centrifugation. Aqueous supernatants were dried by vacuum centrifugation with sample temperature maintained at -4ºC (Labconco, Kansas City MO, USA). Pellets were subsequently resuspended in 25 µl of H_2O as the injection buffer. To better detect some of the deoxy-triphosphate nucleotides, a periodate treatment was performed on the dry cell extract to remove highly abundant triphosphate nucleotides that could interfere with lower abundance metabolites (10). For targeted metabolite analysis and relative concentration determination of metabolites, samples were injected onto an Agilent 6470 Triple Quadrupole (QQQ)-LC-MS/MS. Mass spectrometer was equipped with an Agilent Jet-stream electrospray ionization (ESI) source and samples were analyzed in positive mode. Multiple reaction monitoring (MRM) transitions were optimized on standards for each metabolite. ESI Source gas temperature and flow were set at 350°C and 5 L/min respectively, sheath gas flow and temperature were 250°C and 11 L/min respectively. The nebulizer pressure was set at 45 psi and capillary and nozzle voltage were set at 3500V and 500V. Relative concentrations were determined from external calibration curves. Data were analyzed using MassHunter Quant (Agilent Technologies). For triphosphate nucleotides (ATP, GTP, CTP, ITP and UTP), deoxy-triphosphate nucleotides (dATP, dGTP, dTTP and dUTP) and nucleosides (dC and dU) were resolved using an Agilent 1290 UHPLC system. Chromatographic separation was performed on a Scherzo SM-C18 column 3 µm, 3.0×150mm (Imtakt Corp, JAPAN). The chromatographic gradient started at 100% mobile phase A (5 mM ammonium acetate in water) with a 5 min gradient to 100% B (200 mM ammonium acetate in 20% ACN / 80% water) at a flow rate of 0.4 ml/min. This was followed by a 5 min hold time at 100% mobile phase B and a subsequent re-equilibration time (6 min) before next injection. Samples were maintained at 4ºC and a volume of 5 µl of sample were injected. The column temperatures were all maintained at 10°C. To better detect dUTP, ion pairing chromatography was used with ionization in negative mode, and metabolite separation was achieved by using a Zorbax Extend C18 column 1.8 µm, 2.1 × 150mm^2 with guard column 1.8 µm, 2.1 × 5mm^2 (Agilent Technologies). The chromatographic gradient started at 100% mobile phase A (97% water, 3% methanol, 10 mM tributylamine, 15 mM acetic acid, 5 µM medronic acid) for 2.5 min, followed with a 5-min gradient to 20% mobile phase C (methanol, 10 mM tributylamine, 15 mM acetic acid, 5 µM medronic acid), a 5.5-min gradient to 45% C and a 7-min gradient to 99% C at a flow rate of 0.25 mL min^{-1}. This was followed by a 4-min hold time at 100% mobile phase C. The column was restored by washing with 99% mobile phase D (90% ACN) for 3 min at 0.25 mL min^{-1}, followed by increase of the flow rate to 0.8 mL min^{-1} over 0.5 min and a 3.85-min hold, after which the flow rate was decreased to 0.6 mL min^{-1} over 0.15 min. The

column was then re-equilibrated at 100% A over 0.75 min, during which the flow rate was decreased to 0.4 mL min^{-1}, and held for 7.65 min. One minute before the next injection, the flow was brought back to forward flow at 0.25 mL min^{-1}. The column temperature was maintained at 35°C. Retention times and linear range of detection were obtained by running authentic standard mixes. Repeat injections of authentic standards were performed throughout the run to observe any shifts in retention time or chromatographic quality. The area under the curve for each sample and metabolite was measured and ensured to be below the saturation limit for those metabolites where range curves were available. No corrections or allowances were made for ion suppression effects.

Cdadc1 deficient mice

All mouse work was approved by the IRCM Animal protection committee (Animal use protocols 2015-10, 2017-01) according to the guidelines of the Canadian Council on Animal Care. *Cdadc1*$^{-/-}$ mice were generated at the McGill University transgenic core facility by CRISPR/Cas9 using the same guides and strategy described above for CH12F3 cells. Briefly, the sequences of the two gRNAs targeting *Cdadc1* exon 4 cloned into pX330 (U6-Chimeric_BB-CBh-hSpCas9, Addgene #42230) were PCR amplified using a forward primer containing a T7 promoter sequence (tTAATACGACTCACTATAGGGcaccGCCCAGTATGTAAGTC, OJ1295 for guide 1 and tTAATACGACTCACTATAGGGcaccGCTCTCATTAAGCATG, OJ1299 for guide 2) and a reverse primer binding at the 3' end of the chimeric guide RNA scaffold and polyA tail (AAAAAAGCACCGACTCGGTGCCACTT, OJ1296). PCR products were purified, *in vitro* transcribed and microinjected with Cas9 protein into fertilized eggs of C57B6/N mice at the McGill University transgenic facility. Pups were screened by PCR using oligonucleotides OJ1028 and OJ1029 (**Table S2**). We selected two alleles with 41 and 43 nucleotide deletions within exon 4, which encodes the CDD1, as verified by Sanger sequencing. Both deletions cause a frameshift that would result in a truncated protein shortly afterwards. Exon 4 skipping would equally result in frameshift and premature stop codons and prevent protein expression. Each selected allele was bred to homozygosity to generate the lines *Cdadc1*$^{-/-}$ d41 and d43, respectively. Disruption of *Cdadc1* expression was confirmed by RT-qPCR on RNA from testis, with equally reduced transcript levels in the targeted mice, consistent with nonsense mediated decay of the transcript bearing a premature stop codon. The mice were backcrossed to C57BL6/J (Jackson Labs) for 10+ generations. Lifespan and fertility determinations were performed using cohorts of WT and KO littermate mice obtained from *Cdadc1*$^{+/-}$ crosses. Nude mice (*Nu/Nu/J*, RRID:IMSR JAX:002019) were purchased from Jackson Labs.

Ectopic pancreatic cancer mouse model

All mouse work was approved by the IRCM Animal protection committee (Animal use protocol # 2021-1103 2020-12 JDN and # 2025-1302 / 2024-09 JDN) according to the guidelines of the Canadian Council on Animal Care. The KPC1245 cell line was cultured to 90% maximum confluence and tested negative for mycoplasma by PCR. Male WT C57BL/6 mice aged 8-10 weeks (bred in the IRCM animal facility) were anesthetized with isoflurane and both flanks were shaved. 0.2 x 10^6 KPC cells in 100 µl of PBS were injected subcutaneously into each flank (Right flank = KPC WT, left flank = *Cdadc1* KO) using a Hamilton syringe and a 22S gauge bevel tip needle. Mice were treated with gemcitabine when the tumor volume reached ~80 mm^3 and again 48 h later (100 or 50 mg/kg in 100 µl PBS, i.p. each time). Control mice equally transplanted but not treated were used to compare growth *in vivo*. Mouse weight and tumor size were monitored

periodically. Tumor volume was calculated as [(Length × Width^2)/2]. Mice were euthanized after 4 weeks or upon reaching predetermined clinical endpoints (e.g. >20% weight loss). Tumor tissues were collected fixed in 10% buffered formalin and embedded in paraffin for histological analysis.

Similar experiments were performed in *Cdadc1*$^{-/-}$ mice. Tumors were allowed to develop to ~50-80 mm^3. Mice were treated with 50 mg/kg gemcitabine twice, as above. Mouse weight, tumor size, and general mouse health were followed up daily. *Cdadc1*$^{-/-}$ mice were euthanized upon reaching endpoint (usually ≥20% weight loss) and paired controls were euthanized at the same time for comparing leukocyte populations. Baseline leukocyte populations were determined in untreated mice of similar age.

Analysis of leukocyte populations

BM cells from WT and *Cdadc1*$^{-/-}$ mice were isolated by flushing femur and tibia bones with PBS using a 23G needle. The cells were passed through a 70 µm strainer to obtain a single-cell suspension, washed in PBS, and resuspended in 1.5 mL of red blood cell lysis buffer (155 mM NH4Cl, 10 mM KHCO3, 0.1 mM EDTA) for 5 minutes at RT. After washing, cells were resuspended in PBS with 1% BSA. 4x10^6 cells were stained with antibody combinations for lymphocyte population analysis. Blood samples (100 µL) were collected via retro-orbital bleed into MiniCollect tubes (0.5 mL K3E K3EDTA, Greiner Bio-One, cat. 450475), resuspended in 1.5 mL RBC lysis buffer, and passed through a 70 µm strainer to obtain single-cell suspensions. These were stained for lymphocyte analysis with the same antibody combinations.
Cell proportions in blood and bone marrow were determined by flow cytometry using a combination of antibodies against CD45-APC-Cy7 (clone: 104), CD3-PerCP-Cy5.5 or PE (Clone: 145-2C11) (BD Pharming), Ly6G-Pacific blue (clone: 1A8), CD11b-APC (Clone: M1/70), B220-AlexaFluor 700 (Clone: RA3-6B2), CD34-PE (Clone: MEC14.7) (BioLegend). Data acquisition was performed using BD LSR Fortessa (BD Biosciences) and analyzed with FlowJo (BD Biosciences). Absolute cell counts per mL were calculated using 123count eBeads™ (Invitrogen, cat. 01-1234-42).

Chemogenomic genome-wide screen

The genome-wide pooled CRISPR/Cas9 KO screen made in the presence of gemcitabine was performed by the ChemoGenix platform (IRIC, Université de Montréal; https://chemogenix.iric.ca/), as previously described (11). Briefly, a NALM6 clone bearing an integrated doxycycline-inducible Cas9 expression cassette generated by lentiviruses made from pCW-Cas9 (Addgene #50661) was transduced with the genome-wide KO EKO sgRNA library (278,754 different sgRNAs) (12). After thawing the library from liquid N_2 and letting it recover in 10% FBS RPMI for 1 day, KOs were induced for 7 days in culture with 2 ug/mL doxycycline. The pooled library was then split into different T-75 flasks (28x10^6 cells per flask; a representation of 100 cells/sgRNA) in 70 mL at 4x10^5 cells/mL. Cells were treated with gemcitabine (3nM, using 1000X DMSO stock solution) for 8 days with monitoring of growth every 2 days, diluting back to 4x10^5 cells/mL and adding more compound to maintain same final concentration whenever cells reached 8x10^5 cells/mL. Over that period, treated cells had 5.74 population doublings whereas DMSO-only treated negative controls had 7.03 doublings. Cells were collected, genomic DNA extracted using the Gentra Puregene kit according to manufacturer's instructions (QIAGEN), and sgRNA sequences PCR-amplified as described (12). sgRNA frequencies were obtained by next-generation sequencing (Illumina NextSeq 500). Reads were aligned using Bowtie2.2.5 in the

forward direction only (–norc option) with otherwise default parameters and total read counts per sgRNA tabulated. Context-dependent chemogenomic interaction scores were calculated using a modified version of the RANKS algorithm (12), which uses guides targeting similarly essential genes as controls to distinguish condition-specific chemogenomic interactions from non-specific fitness/essentiality phenotypes. Raw read counts are available upon request from the ChemoGenix platform.

Western blotting

For direct western blotting, 2 x 10^6 cells were harvested and washed in PBS. Cell pellets were suspended in 40 µl RIPA lysis buffer [50 mM Tris-HCl pH 8.0, 150 mM NaCl, 1% NP-40, 5 mM EDTA, 0.5% [w/v] Sodium deoxycholate, 0.1% [w/v] SDS, 1X Complete protease inhibitors (Roche)] and incubated with gentle rotation for 30 min at 4°C. The cell suspension was sonicated 10 min on ice and cleared by centrifugation 10 min at 13,000 rpm. The cell extract was re-suspended in 6 µl of Laemmli buffer 5X [250mM Tris-HCl pH6.8, 10%SDS, 50% glycerol, 0.1% [w/v] bromophenol blue, 5% b-Mercaptoethanol] and boiled for 10 min. After centrifugation at 10,000 x g, 5 min, RT, the supernatant was run in SDS-PAGE and electroblotted onto nitrocellulose (Bio-Rad). Membranes were probed with mouse monoclonal anti-CDADC1 (Abnova #H0081602-M01, 1:1000 dilution), mouse monoclonal anti-DCTD (Santa Cruz, #SC-375699, 1:500 dilution), rabbit monoclonal anti-DUT (Abcam # ab137102, 1:1000 dilution), rabbit polyclonal anti-SAMHD1 (Proteintech #12586-1-AP, 1:1000 dilution), anti-Actin (Sigma #A2066), anti-tubulin (DSHB #E7).

Immunoprecipitation

To detect endogenous CDADC1 protein, a concentration step by immunoprecipitation was used as follows: 15-20 x 10^6 cells were harvested and washed in PBS. Cell pellets were suspended in 750 µl NP-40 lysis buffer [20 mM Tris-HCl pH 8.0, 137 mM NaCl, 10% Glycerol, 2 mM EDTA, 1% NP-40, 1X Complete protease inhibitors (Roche)] and incubated with gentle rotation for 30 min at 4°C. The cell suspension was sonicated 10 min on ice and cleared by centrifugation 10 min at 13,000 rpm. Anti-CDADC1 rabbit polyclonal antibody (28 µg) (Abnova #H00081602-D01 lot D9031) was incubated with 40 µl Protein A-Sepharose 4B beads (Invitrogen) in 100 µl lysis buffer at 4°C for 4 h. The bead-antibody conjugates were spun down at 2,000 x g for 2 min at 4°C and washed 3 x with lysis buffer containing 0.5x Complete protease inhibitors. The cell extract was added to the beads-antibody complex and incubated overnight at 4°C, then washed 3 times with lysis buffer containing 1X Complete protease inhibitors. The beads were re-suspended in 40 µl 2X SDS loading buffer [0.4M Tris-HCl pH6.8, 10%SDS, 50% glycerol, bromophenol blue, 100 mM DTT] and boiled for 10 min. After centrifugation at 10,000 x g, 5 min, RT the supernatant was run in SDS-PAGE and electroblotted onto nitrocellulose (Bio-Rad). Membranes were probed with anti-CDADC1 mouse monoclonal (Abnova #H0081602-M01, 1:1000 dilution).

Immunohistochemistry:

Section of 5-µm paraffin-embedded tissues were deparaffinized in xylene (3 changes, 5 min each) and rehydrated in distilled water using graded alcohols (3 min each). Antigen retrieval was performed by steaming slides for 20 min, followed by cooling in Citrate-EDTA buffer (10 mM Citric Acid, 2 mM EDTA, 0.05% Tween 20, pH 6.2) for 20 min. Endogenous peroxidase was blocked with 0.3% hydrogen peroxide for 10 min, and endogenous biotin was blocked for 15 min using the

Avidin/Biotin System (Vector Laboratories, #SP2001). For Caspase 3, an additional permeabilization step with 0.2% Triton was performed. Protein blocking was done with 10% normal goat serum and 1% BSA for 60 min at RT. Sections were incubated with anti-CD3 (1:100, rabbit Mab, Abcam, #ab5690) and anti-Caspase 3 (1:100, Cleaved Caspase-3 (Asp175) (5A1E) Rabbit mAb, Cell Signaling, t#9664S) for 60 min at RT. Biotin-conjugated secondary antibodies (goat anti-rabbit IgG, 1:200, Vector Laboratories) were detected using the Vectastain ABC kit (Vector Laboratories, PK-6100). Peroxidase activity was developed with ImmPACT NovaRED HRP substrate (Vector Laboratories). Sections were counterstained with hematoxylin (Sigma, #MHS32-1L) for 2 min and mounted.

Histological analysis

Slides were blindly evaluated by a board-certified pathologist. CD3-positive and Caspase 3-positive individual cells were counted in specific regions for up to 10 subsequent high-power field (HPF) when possible (400X magnification, Leica DM3000, ocular HC PLAN 10X/25) and the mean number of cell/HPF was calculated. When not possible to count in 10 HPF (smaller tumors), the maximum number of fields was counted and the mean calculated accordingly. Data were presented as paired as to account for interindividual variations (one WT and CDADC1 KO tumor per biological replicate). Regions evaluated for CD3-positive cells included the tumor parenchyma and margin, whereas Caspase-3 cells were counted in the tumor parenchyma. Necrotic regions were excluded from the evaluation in Caspase-3 slides to limit potential bias associated with central necrosis in larger tumors.

RT-qPCR

RNA was isolated from testis using TRIzol (Life Technologies), following manufacturer's instruction, and quantified by NanoDrop (ThermoFisher). cDNA was synthesized from 1 µg of RNA using the ProtoScript^{TM} M-MuLV Taq RT-PCR kit and random primers (New England BioLabs). Quantitative PCR using SYBR select master mix (Applied Biosystems) was performed and analyzed in a ViiATM 7 machine and software (Life technologies). Primers for quantitative PCR were designed with NCBI RNA blast and synthesized at Integrated DNA technologies. Primers are listed in **Table S2**.

Phylogenetic analysis.

Multiple protein sequences obtained from GenBank were aligned using Clustal Omega 1.2 and uploaded into MEGA (Molecular Evolutionary Genetics Analysis) 6.0.5 (13). The proportion of site-specific amino acid differences was calculated using the p-distance substitution model. Unrooted phylogenetic trees were constructed by the neighbor-joining method (14). The reliability of the topology of the neighbor-joining tree was tested with Felsenstein's bootstrap method (15) and replicated 2000 times. The following protein sequences built the tree in Fig. 1: *Ceratotherium simum simum* (APOBEC1: XP_004438714); *Danio rerio* (ADAR1: NP_571671; ADAR2: NP_571685; ADAT1: NP_001076356; ADAT2: XP_005160675; AID: NP_001008403; APOBEC2: NP_001161935; CDA: NP_991242; CDADC1: NP_001007449; DCTD: NP_001017639); *Gallus gallus* (ADAR2: NP_989571; ADAT1: NP_001012797; ADAT2: XP_419709; AID: NP_001230151; APOBEC2: XP_418038; CDA: NP_001139516; CDADC1: XP_417059; DCTD: NP_001006444); *Homo Sapiens* (ADAR1: NP_001102; ADAR2: NP_056648; ADAT1: NP_036223; ADAT2: NP_872309; AID: NP_065712; APOBEC1: NP_001635; APOBEC2:

NP_006780; APOBEC3C: NP_055323; APOBEC3D: NP_689639; APOBEC3F: NP_660341; CDA: NP_001776; CDADC1: NP_112173; DCTD: NP_001012750); *Mus Musculus* (ADAR1: NP_001139768; ADAR2: NP_001020008; ADAT1: NP_038953; ADAT2: NP_080024; AID: NP_033775; APOBEC1: NP_001127863; APOBEC2: NP_033824; APOBEC3: NP_001153887; CDA: NP_082452; CDADC1: NP_082262; DCTD: NP_848903); *Myotis lucifugus* (APOBEC1: XP_006084271); *Pleurodeles waltl* (AID: CBG76579); *Sarcophilus harrisii* (ABOBEC1: XP_003771254); *Taeniopygia guttata* (ADAR1: ENSTGUT00000004281); *Xenopus laevis* (APOBEC2: NP_001086311; DCTD: NP_001084625); Xenopus tropicalis (ADAR1: XP_002943575; ADAR2: NP_001096190; ADAT1: NP_001037984; ADAT2: NP_001072562; CDA: NP_001017217; CDADC1: XP_004912022). Sequence logos were generated using Weblogo (weblogo.berkeley.edu/logo.cgi) (16) from the alignment of 18 vertebrate species spanning from cartilaginous fishes to man (*Callorhinchus milii*, XP_007889736; *Danio rerio*, NP_001007449, *Takifugu rubripes*, XP_003966473, *Xenopus tropicalis*, XP_004912022; *Gallus gallus*, ENSGALT00000027467; *Taeniopygia guttata*, XP_002198216, *Alligator sinensis*, XP_006030262; *Chrysemys picta bellii*, XP_005287191, *Chelonia mydas*, XP_007072422; *Ornithorhynchus anatinus*, XP_001513940; *Monodelphis domestica*, XP_001378718, *Sarcophilus harrisii*, XP_003764620; *Dasypus novemcinctus*, XP_004478276, *Eptesicus fuscus*, XP_008143821, *Orcinus orca*, XP_004274652, *Loxodonta Africana*, XP_003412614, *Canis lupus familiaris*, XP_534116, *Mus musculus*, NP_082262; *Homo sapiens*, NP_112173.

Gene expression and gene alterations data mining

The data for bulk human tissue gene expression by RNA-seq was plotted at the GTEx Portal (gtexportal.org), using GTEx analysis release V8 (dbGaP Accession phs000424.v8.p2). Additional bulk RNA-seq data from human tissues and cell lines was obtained from the Human protein atlas (proteinatlas.org) (17). Gene expression RNA-seq data from the Cancer cell line Encyclopedia version Broad Institute 2019, and from The Cancer Genome Atlas (TCGA) were analyzed and plotted at cBioportal (cbioportal.org) using default parameters (18). TCGA gene expression data compared to unpaired normal human tissue samples was performed by the GEPIA 2 gene expression profiling server (gepia2.cancer-pku.cn) (19). Survival analysis of TCGA data as a function of CDADC1 expression was obtained from OncoDB (oncodb.org, August 2021 data release) (20). Microarray Innovations in LEukemia (MILE) study dataset (GSE13159) was analyzed in the GEO database with GEO2R using GLP570 probes. Values are the mean of 4 probes for CDADC1 in the Affymetrix Human Genome U133 Plus 2.0 Array 4 were averaged. Data is provided as log2-transformed, normalized expression values to facilitate comparison across samples and conditions (21). CDADC1 variants were enumerated from the gnomAD v4.1.0 (22) (gnomad.broadinstitute.org), and ClinVar database (www.ncbi.nlm.nih.gov/clinvar/). *CDADC1* copy number alterations and mutation in cancer were determined at cBioPortal, using a curated set of 226 non-redundant studies totaling 101480 patients. The data was filtered for studies with ≥50 patients and a minimum of 1% alterations in *CDADC1*.

Statistical analyses

Statistical tests and P-values for statistically significant differences are indicated in the corresponding figure legends and text. No data was excluded from analyses. All datapoints plotted in the figures represent biological replicates. For experiments with cell lines biological replicates were defined as the same cell line or clone measured in independent experiments, as well as independently generated clones measured in the same experiment. All statistical tests

were performed with Prism (Graphpad). Groups and datasets were tested for normality using the Shapiro-Wilk and D'Agostino-Pearson tests. Unpaired two-tailed t-test with Welch’s correction was used to compare any 2 groups. For two variables, 2-way ANOVA was used, with post-hoc Tukey's method to compare every mean to every other mean, and Bonferroni’s method to compare between specific groups. One-way ANOVA with Dunnett's was used for multiple comparisons.

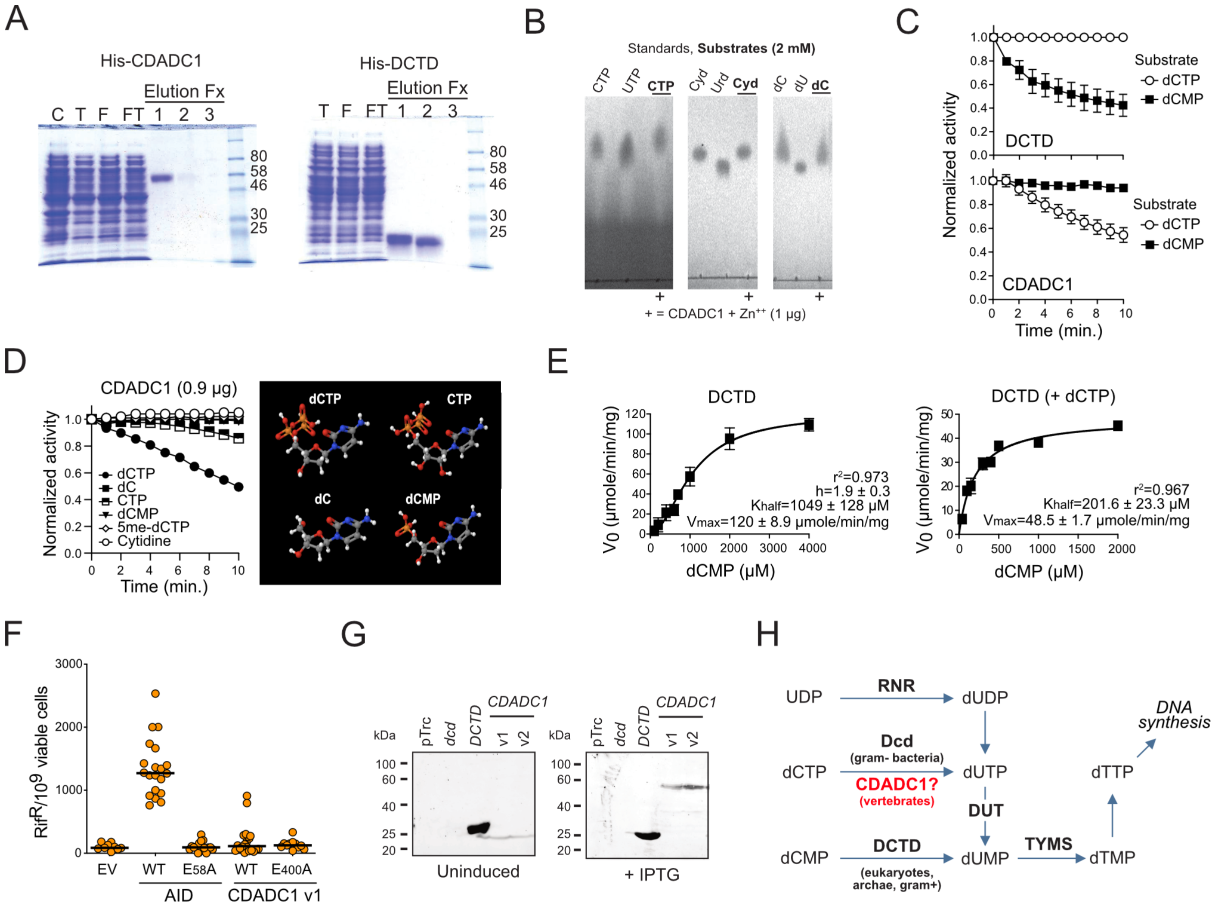

Figure S1 – Characterization of CDADC1 enzymatic activity

A) Coomassie blue-stained SDS-PAGE of representative recombinant human CDADC1v1 and DCTD purification from *E. coli dcd*. T, total; C, clarified; and F, filtered lysates; FT, flow through; Elution Fx, fractions eluted from nickel column.

B) TLC plates resolving reactions after incubating CDADC1 with CTP, cytidine (Cyd) or deoxycytidine (dC) for 15 min at 37˚C. Standards (10 µg) for each substrate and expected deamination product run in parallel.

C) Deamination activity of DCTD and CDADC1 on the indicated substrates, monitored by decrease in absorbance at 290 nm over time. Normalized mean ± SEM of 3 reactions with independent protein preparations.

D) Substrate specificity of CDADC1 monitored by Abs 290 nm. Normalized mean ± SEM from 2-3 reactions with independent enzyme preparations. A comparison of the conformation of dC and its nucleotides is shown to the right.

E) Plots of DCTD initial velocity (V_0) calculated as a function of substrate concentration in reactions with 350 ng His-DCTD in the absence or presence of 0.1 mM dCTP (allosteric activator). Mean ± SEM with best model fit from 2 reactions with independent protein preparations. Enzyme kinetics parameters obtained from the curves are indicated.

F) Fluctuation assay monitoring the frequency of rifampicin-resistant (Rif^R) *Escherichia coli* colony forming units (c.f.u.) when expressing pTrcHisA, vector empty vector (EV) or encoding the indicated enzymes. Each symbol indicates the frequency of Rif^R c.f.u per 10^9 viable c.f.u. in an independent culture. Data compiles four experiments for each variant, with median values (horizontal lines). Significant P-values by Kruskal–Wallis test with Dunnet multiple test comparison (α<0.05).

G) Western blots of total extracts of *E. coli* BW1105 transformed with pTrcHis empty (pTrc) or encoding the indicated enzymes before and after IPTG induction, probed with anti-His antibody. The leaky DCTD expression explains growth complementation in minimal medium in the absence of IPTG in Fig 2A.

H) Scheme of the three known pathways to synthesize dUMP as substrate for thymidylate synthase (TYMS), and their species distribution. Relevant enzymes are indicated (RNR, ribonucleotide reductase; DUT, deoxyuridine triphosphatase).

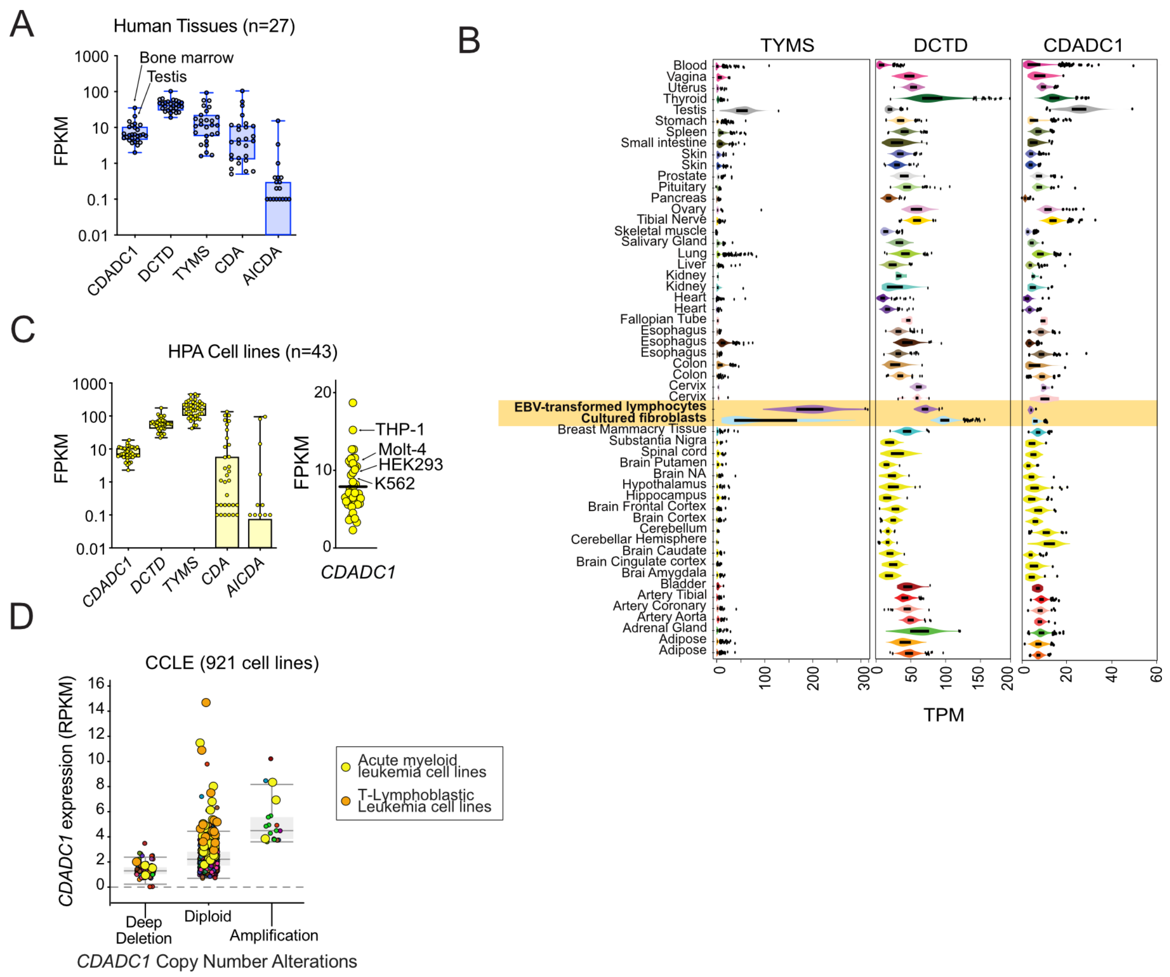

Figure S2 – CDADC1 gene expression

A) *CDADC1* and *DCTD* transcript levels in Fragments Per Kilobase per Million mapped reads (FPKM) across 27 human tissues. Tissue-specific *AICDA* (expressed largely in lymph node) provides an example of a gene not expressed in most tissues. RNA-seq data from the Human Protein Atlas.

B) Violin plots compiling RNA-seq transcript per million (TMP) for *CDADC1*, *DCTD*, and *TYMS* across human tissues and cells. Samples per tissue range from 30 to 430, except cervix (n=6), bladder (n=11), and fallopian tubes (n=6). Rapidly dividing cultured fibroblasts and EBV-transformed lymphoblastoid cells are highlighted. Data analyzed and plotted at GTEx Portal.

C) Expression levels of the indicated genes in 44 human cells lines. Boxes indicate 5-95% with median values. The scatter plot on the right shows the individual values for *CDADC1* in each cell line in a linear scale, highlighting cell lines relevant to this work. Data \ from the Human Protein Atlas site.

D) Expression of *CDADC1* in 921 human cell lines stratified by copy number alterations of the chromosome 13 region containing the *CDADC1* locus. Cell lines from 42 different origins (organs, tissues, cell types) are color coded. For simplicity we only highlight the “Leukemia” and “T-lymphoblastic leukemia/lymphoma” categories. Data from the cancer cell line encyclopedia.

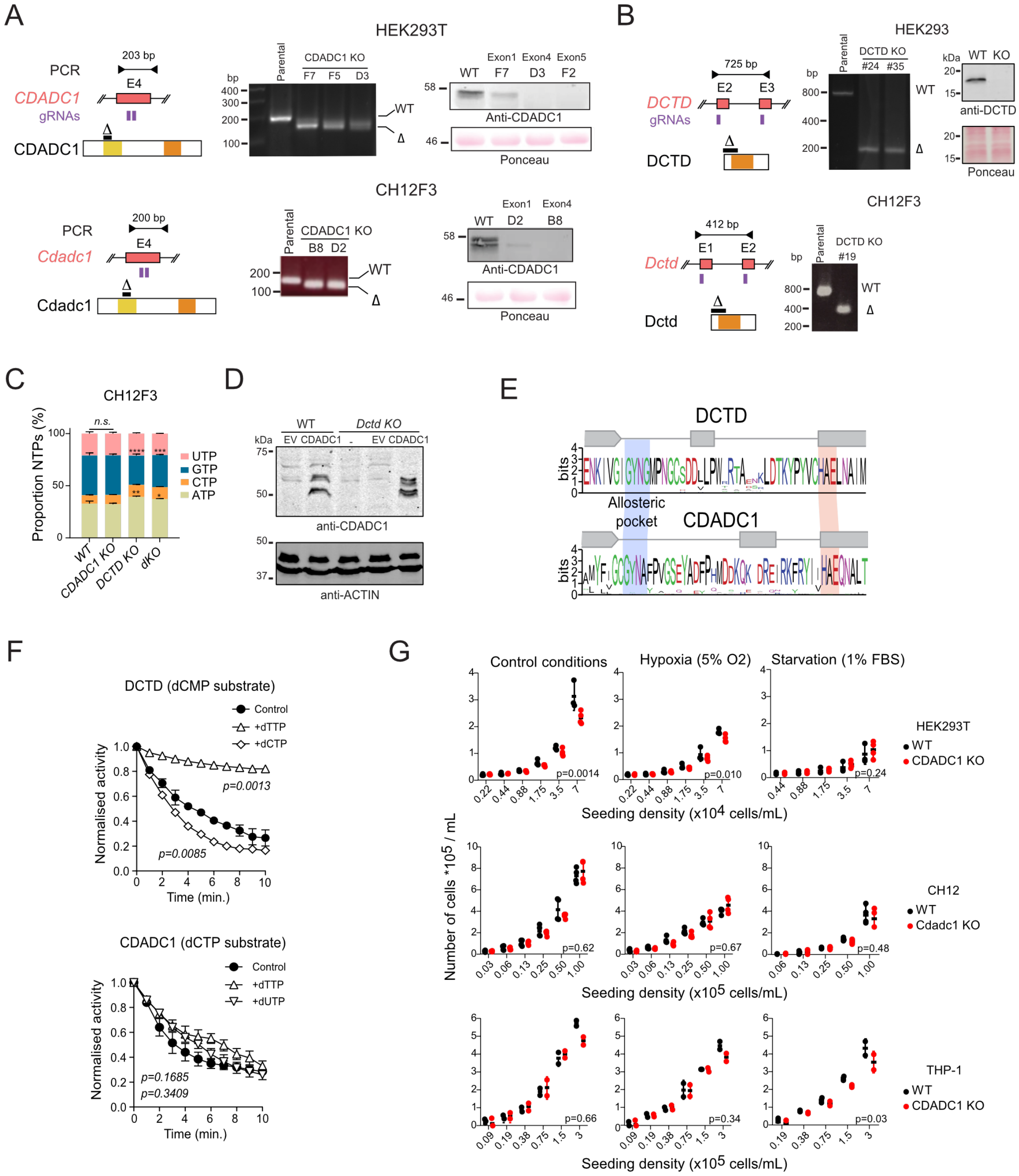

Figure S3 – Genetic ablation of CDADC1 and DCTD and its effect on cell growth

A) Main strategies for *CDADC1* and *Cdadc1* inactivation in human and mouse cell lines by introducing a deletion in exon 4 (E4) using CRISPR-Cas9 with two gRNAs. Genotyping PCR with WT fragment size and a representative agarose gel with WT and several targeted clones in human HEK293T and mouse CH12F3 cell lines are shown (middle panels). Representative western blots of the protein immunoprecipitated from 2 x 10^7 cell equivalents extracts using rabbit polyclonal anti-CDADC1 antibody and blotting with anti-CDADC1 mouse monoclonal antibody are shown for parental HEK293T and CH12F3 cells and targeted single cell clones, indicating the targeted exon in each case (see detailed methods for additional details on each strategy). Targeting exon 1 enabled some residual protein production, while targeting exons 4 or 5 fully prevented protein expression even in clones with in-frame deletions (e.g., HEK293T clone D3).

B) Strategy for inactivating *DCTD/Dctd* in human and mouse cell lines. Genotyping PCR with WT fragment size and a representative agarose gel with WT and several targeted clones in HEK293T and CH12F3 cells are shown. Western blot shows loss of protein expression with this strategy in HEK293T cells. This antibody does not recognize mouse Dctd.

C) Relative proportion of NTPs by LC-MS/MS in CH12F3 cells**.** Mean + SEM of 5 biological replicates per genotype. Asterisks indicate p-values (**** $p<0.0001$, *** $p=0.0001$, ** $p=0.001$, * $p<0.0295$) for significant differences versus WT cells from 2-way ANOVA with Dunnett multiple comparison test (all versus WT). WT versus CDADC1 KO not significantly (n.s.) different by 2-way ANOVA with Bonferroni's multiple comparisons test.

D) Western blot of whole CH12F3 cell extracts for WT and *Dctd* KO transduced with pSLIK empty (EV) or encoding \ CDADC1v1, 72 h after induction with 100 ng/ml doxycycline. Probed with anti-CDADC1 MAb, which does not detect endogenous CDADC1.

E) Sequence logo of the DCTD region containing the allosteric pocket (in blue) upstream from the active site (in red), aligned to the conserved region in CDADC1.

F) Activity of DCTD on dCMP or CDADC1 on dCTP, monitored by absorbance at 290 nm, without or with 0.5 mM dCTP (allosteric activator) or dTTP (allosteric inhibitor). Normalized mean ± SEM of 3 reactions with independent protein preparations for each enzyme. P-values for significant differences from 2-way ANOVA with Tukey's test.

G) Comparison of cell growth under chronic hypoxic or starvation conditions. Cells were plated at different densities in their normal medium supplemented with either 10% FBS and incubated at 37˚C in CO_2 incubator at atmospheric O_2 levels (~18-21% O_2) (Control) or in a hypoxia chamber (5% O_2) or supplemented with 1% FBS (starvation) and incubated as controls. Live cells were measured after 48 h. P-values from 2-way ANOVA test.

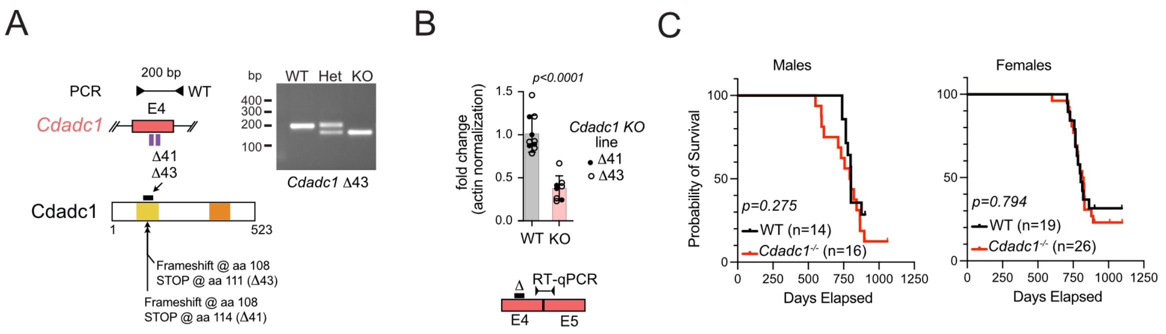

Figure S4 – Generation and characterization of Cdad1-deficient mice

A) Strategy to inactivate *Cdadc1* in mice. Two independent lines of *Cdadc1*$^{-/-}$ mice with 41 or 43 bp deletions in exon 4 were generated. Genotyping PCR design and representative agarose gel are shown.

B) *Cdadc1* transcript levels in testis from WT and *Cdadc1*$^{-/-}$ lines quantified by RT-qPCR is shown to the right. P-value by two-tailed unpaired t-test.

C) Kaplan-Meier survival plot of WT versus *Cdadc1*$^{-/-}$ littermate mice segregated by sex. P-values from long-rank test.

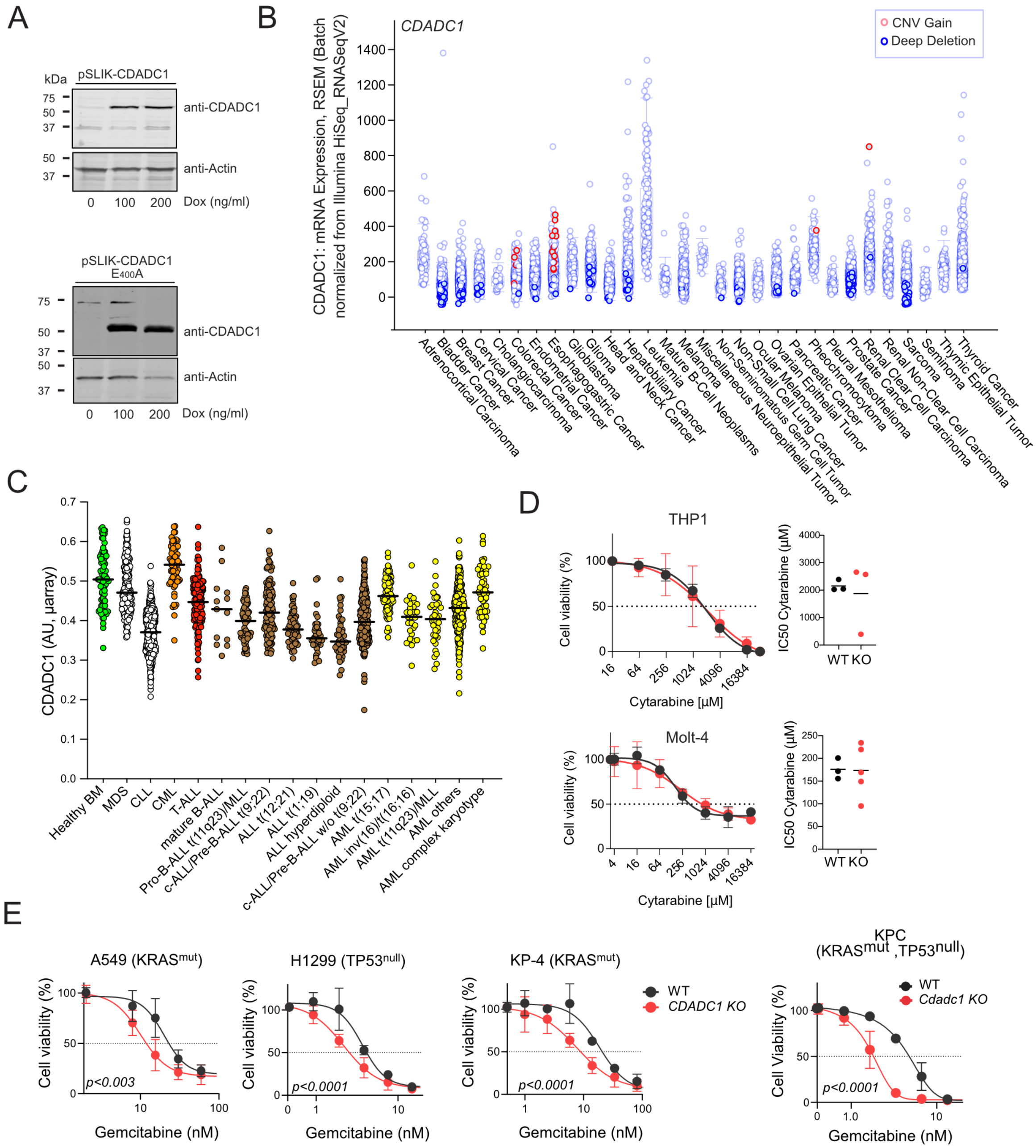

Figure S5 – CDADC1 expression and dC analog sensitivity in human cancer cells

A) Western blot of whole HEK293T cell extracts transduced with pSLIK encoding human CDADC1v1 WT or catalytically inactive $E_{400}A$ version, 72 h post-induction with 100 ng/ml doxycycline. Probed with anti-CDADC1 MAb. Endogenous CDADC1 is not detected.

B) *CDADC1* transcript levels across human cancer types. RNA-seq and copy number variations (CNV) data from TCGA analyzed and plotted in cBioportal. Samples with deep deletions encompassing *CDADC1* locus and copy number gains are highlighted.

C) *CDADC1* transcript levels in different types of leukemia. Microarray data from the MILES project (GSE13159).

D) Relative cell viability of WT and *Cdadc1* KO variants of human leukemia cell lines grown in the presence of cytarabine for 72 h. Curves show mean ± SD of 3 independent experiments. Scatter plot shows calculated IC_{50} for biological replicates.

E) Relative cell viability of WT and *CDADC1 KO* human epithelial cancer cell lines or WT and *Cdadc1 KO* KPC mouse pancreatic cancer cell line grown in the presence of gemcitabine and after 72 h. Mean ± SD of 2 (KP-4) or 3 experiments with 3 independent KO clones per cel line.

D,E) Significant P-values from 2-way ANOVA test.

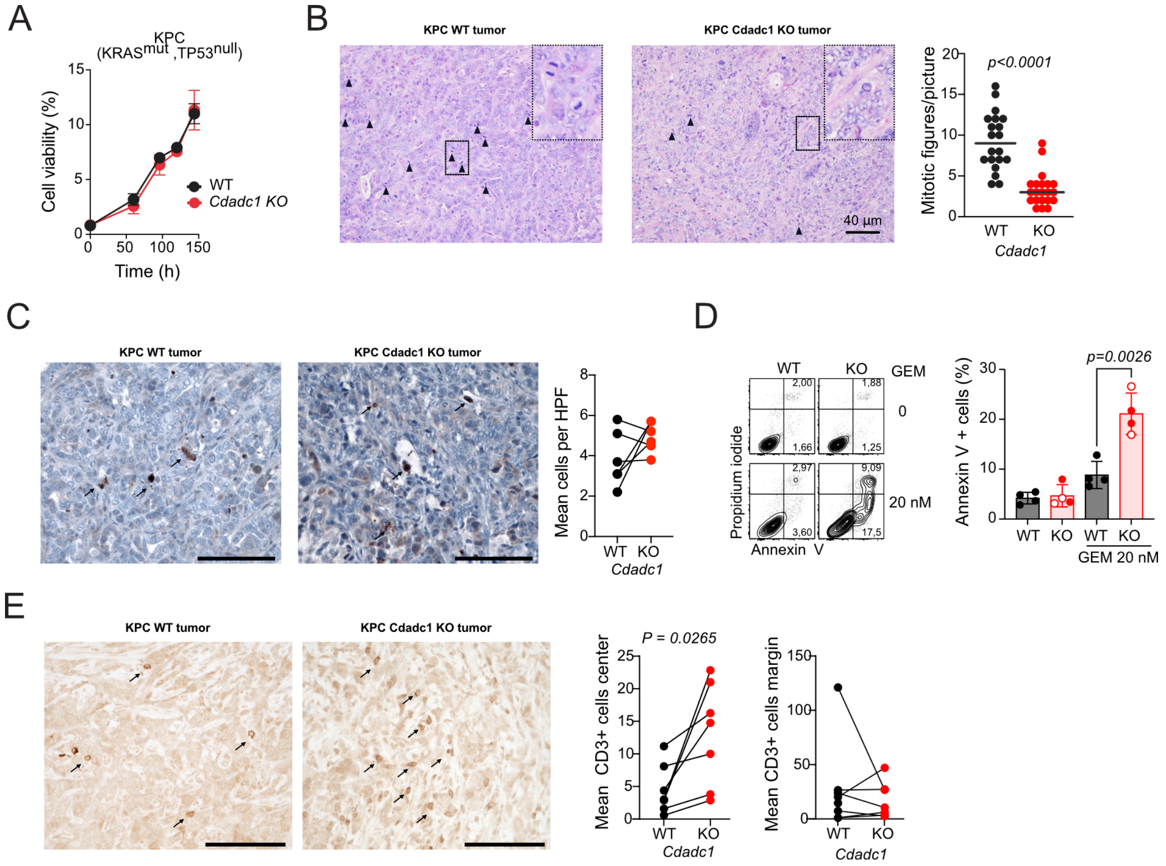

Figure S6 – Immunohistochemical analysis of KPC WT and *Cdadc1* KO tumors.

A) Growth curve of KPC WT and *Cdadc1* KO cells by cell enumeration. Mean ± SEM. for 3 experiments.

B) Representative micrographs of hematoxylin & eosin staining in paired WT and *Cdadc1* KO KPC tumors obtained from C57BL6/J mice the end of gemcitabine treatment. Arrowheads indicate mitotic figures. Magnification 20X, bar = 40 µm. Mitotic figure quantification from 4 tumors (5 pictures/tumor) per genotype, with mean values (bars).

C) Representative immunohistochemical image of cleaved Caspase 3-positive cells or apoptotic debris (black arrows) in the center of WT and *Cdadc1* KO tumors. Counterstained with hematoxylin. Bar=100µm. Quantification was performed on WT and KO tumors obtained from the same mouse. Data are presented as paired.

D) Representative flow cytometry plots of Annexin V and propidium iodide (PI) staining to monitor apoptosis in KPC WT and *Cdadc1* KO treated with 20 nM gemcitabine. The plot shows mean (bars) ± SD of 4 biological replicates (symbols) from 2 independent experiments with 2 clones each.

E) Representative immunohistochemical image of CD3-positive cells (black arrows) in the center of WT and *Cdadc1* KO tumors. Mean CD3-positive cells number per high power field (HPF) (400X magnification) were scored from tumors center and margin. Quantification was performed on WT and KO tumors obtained from the same mouse. Data are presented as paired.

B-E) P-value for significant differences from unpaired (B,D) or paired (C,E) two-tailed t-test.

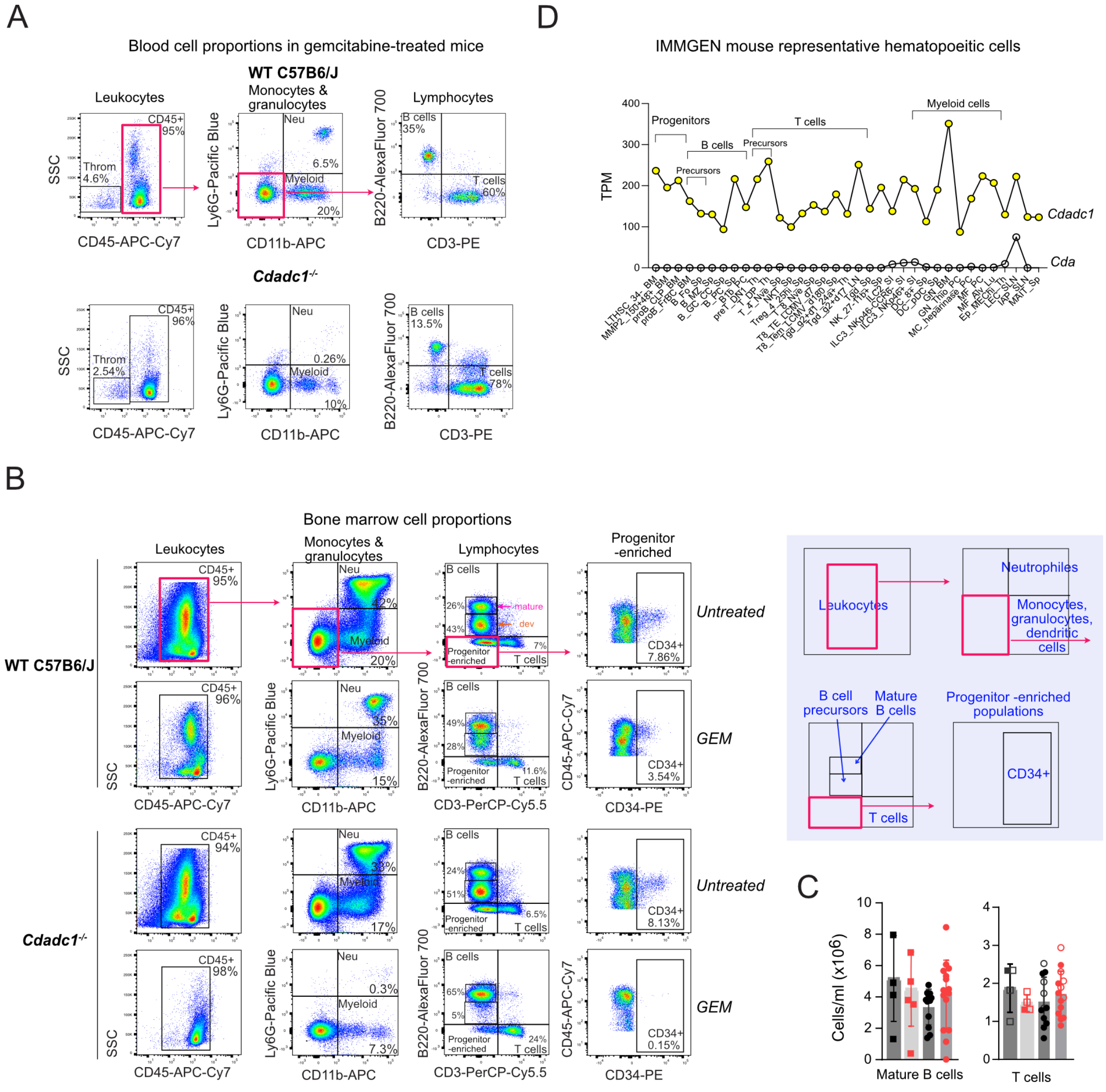

Figure S7 – Gemcitabine toxicity in Cdadc1-deficient mice.

A) Representative flow cytometry plots of blood leukocyte populations in mice treated with gemcitabine. Gating used for the analysis is indicated on the top.

B) as in A), for bone marrow populations of mice untreated and treated with gemcitabine (GEM), with population gating definition.

C) Cell counts in bone marrow for recirculating mature B cell and T cells (quiescent cell populations) calculated from data in B). No statistical differences by one-way ANOVA with Tukey's multiple comparison.

D) Transcript per million (TPM) values for *Cdadc1* and *Cda* across representative hematopoietic cell types from the Immunological Genome Project (ImmGen) database. Data are plotted as means from the ImmGen dataset. Different cell types from bone marrow (BM), spleen (Sp), peritoneal cavity (PC), thymus (Th), small intestine (SI), lymph node (LN).

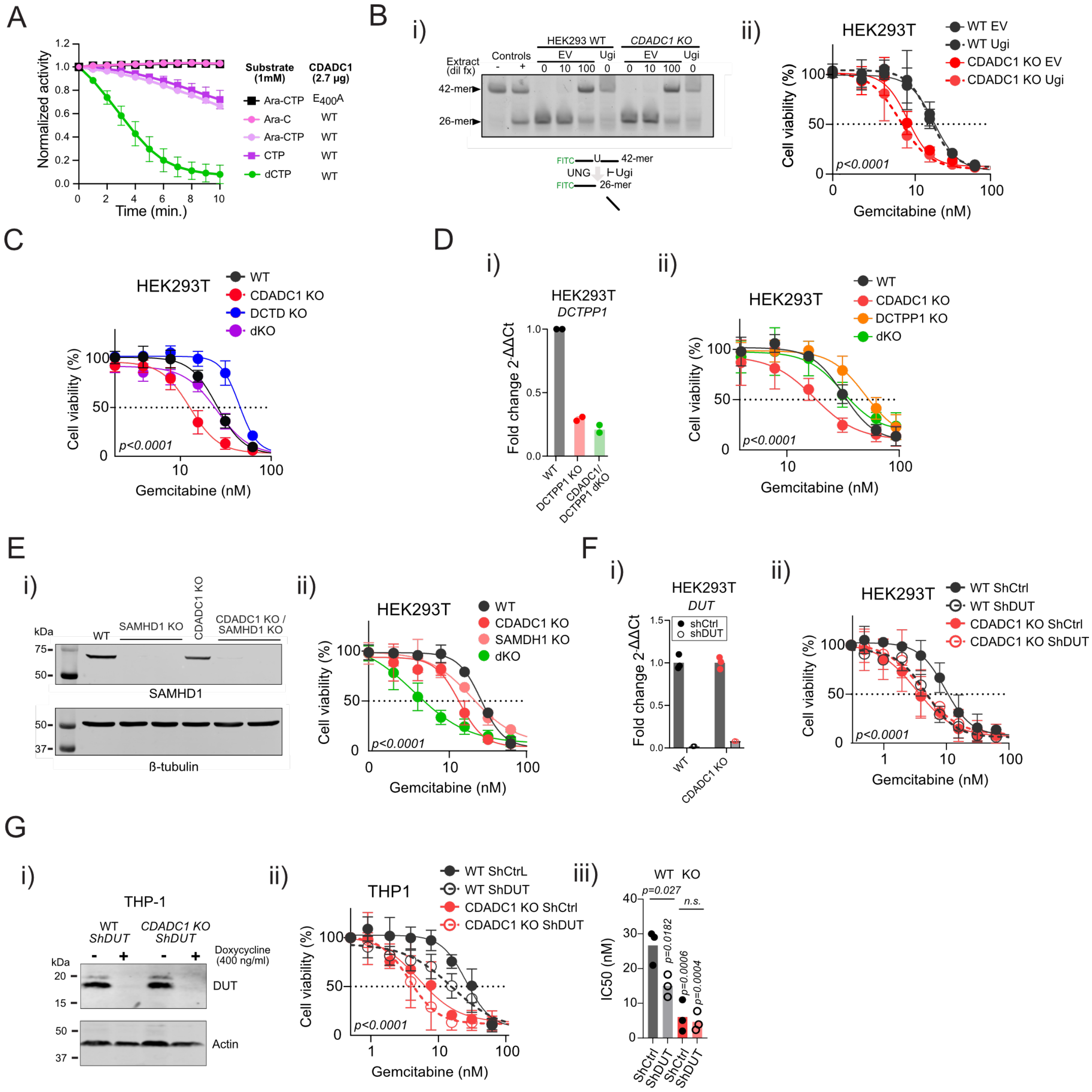

Figure S8 – Effect and mechanism of action of CDADC1 on gemcitabine and decitabine

A) Activity of recombinant CDADC1 on cytarabine nucleoside (ara-C) or triphosphate (ara-CTP), compared to dCTP and CTP, monitored by decrease in absorbance at 290 nm. Normalized mean ± SEM from 3 reactions with independent protein preparations per condition.

B) ***i)*** Gel for UNG activity in cell extracts of HEK293T WT and *CDADC1* KO cells expressing a lentiviral empty vector (EV) or encoding Ugi, measured by the assay illustrated. Substrate and product were separated by TBE-urea acrylamide gel. Extracts dilution factor (dx) is indicated. Controls were substrate alone (-) and incubated with *E. coli* UDG (+). ***ii)*** Survival of HEK293T WT and *CDADC1* KO cells expressing lentiviral empty vector (EV) or encoding the UNG inhibitor Ugi grown in the presence of gemcitabine for 72 h. Mean ± SD from 4 experiments. P-value from two-way ANOVA with Tukey post-test.

C) Survival of HEK293T WT, *CDADC1* KO, DCTD KO, or double KO cells grown in the presence of gemcitabine for 72 h. Mean ± SD from 2 experiments.

D) Survival of HEK293T WT, *CDADC1* KO, *DCTPP1* KO, or double KO cells grown in the presence of gemcitabine for 72 h. Mean ± SD from 3 experiments.

E) ***i)*** Western blot of whole cell extracts from HEK293T WT, CDADC1 KO, SAMHD1 KO, or double KO cells probed with antibodies against SAMHD1 and b-tubulin as loading controls. . ***ii)*** Survival of HEK293T WT, *CDADC1* KO, *SAMHD1* KO, or double KO cells grown in the presence of gemcitabine for 72 h. Mean ± SD from 3 experiments.

C,D,E) P-values from two-way ANOVA with Dunnet's post-test.

F) ***i)*** RT-qPCR demonstrating depletion of DUT in HEK293T cells expression shControl or shDUT. ***ii)*** Survival of HEK293T WT and *CDADC1* KO cells expressing inducible shRNA control or targeting *DUT* grown in the presence of gemcitabine for 72 h. Mean ± SD from 4 experiments. P-value from two-way ANOVA with Tukey post-test.

G) ***i)*** Western blot of whole THP-1 cell extracts expressing doxycycline inducible shRNA control or shRNA against DUT probed with anti-DUT antibody. ***ii)*** Survival of THP-1 WT and *CDADC1* KO cells expressing shRNA control or to DUT, all induced with doxycycline, grown in the presence of gemcitabine for 72 h. Mean ± SD from 3 experiments. ***iii)*** IC_{50} for decitabine calculated from the data in *ii)*. Biological replicates (symbols) and means (bars). Curves show P-value from two-way ANOVA with Tukey's post-test. Scatter plot shows vertical P-values from one-way ANOVA with Tukey's post-test (every condition versus WT shControl condition) and horizontal P-values from unpaired two-tailed t-test for relevant pair-wise comparisons.

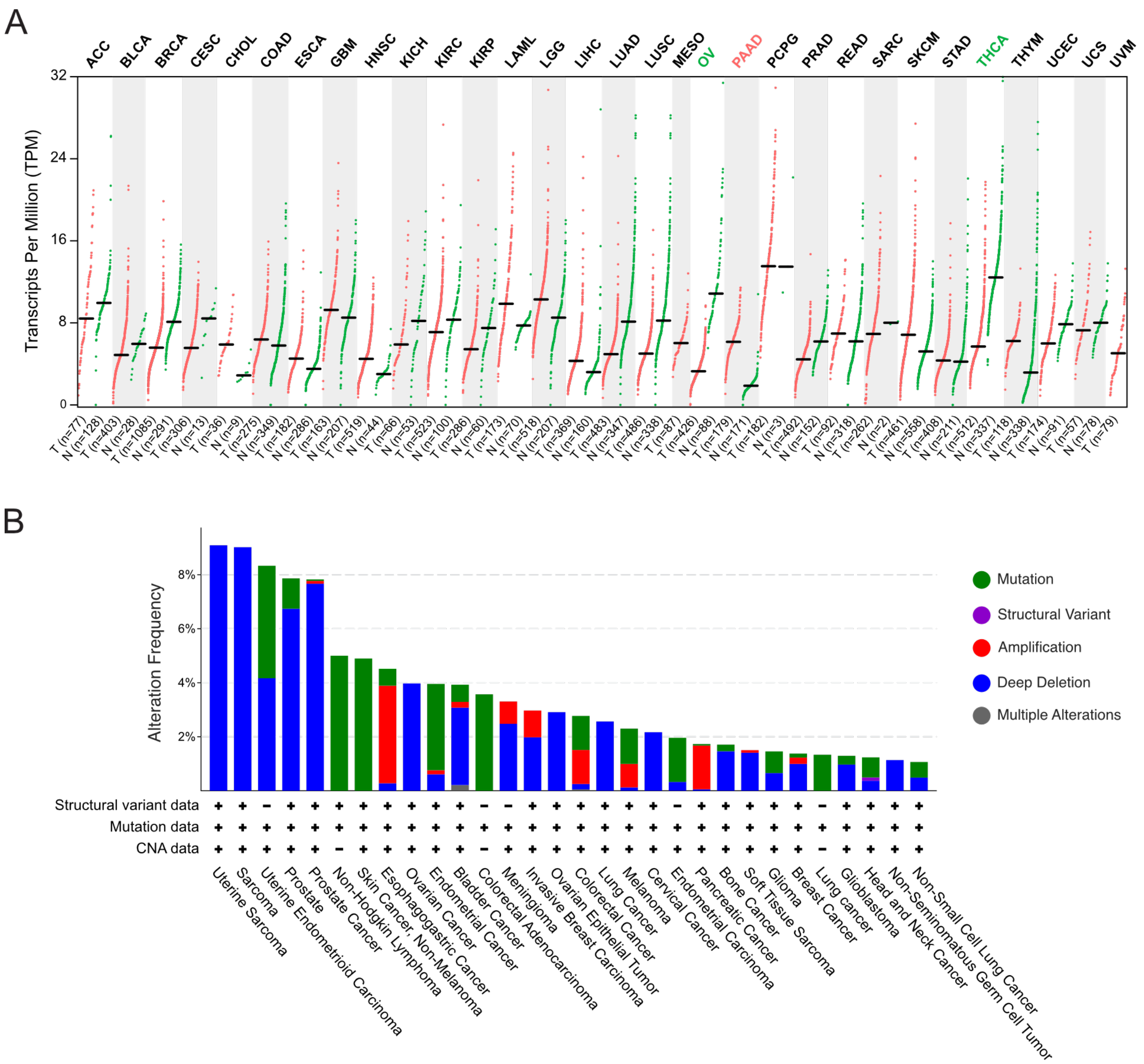

Figure S9 – Expression of CDADC1 transcripts in human cancer and normal tissue

A) *CDADC1* expression in cancer samples from TCGA and normal tissues from GTEx (not paired samples), as provided by the GEPIA 2. Significantly lower (green) and higher (red) expression in tumor versus normal are indicated. *ACC: Adrenocortical carcinoma, BLCA: Bladder Urothelial Carcinoma, BRCA: Breast invasive carcinoma, CESC: Cervical squamous cell carcinoma and endocervical adenocarcinoma, CHOL: Cholangio carcinoma, COAD: Colon adenocarcinoma, DLBC: Lymphoid Neoplasm Diffuse Large B-cell Lymphoma, ESCA: Esophageal carcinoma, GBM: Glioblastoma multiforme, HNSC: Head and Neck squamous cell carcinoma, KICH: Kidney Chromophobe, KIRC: Kidney renal clear cell carcinoma, KIRP: Kidney renal papillary cell carcinoma, LAML: Acute Myeloid Leukemia, LGG: Brain Lower Grade Glioma, LIHC: Liver hepatocellular carcinoma, LUAD: Lung adenocarcinoma, LUSC: Lung squamous cell carcinoma, MESO: Mesothelioma, OV: Ovarian serous cystadenocarcinoma, PAAD: Pancreatic adenocarcinoma, PCPG: Pheochromocytoma and Paraganglioma, PRAD: Prostate adenocarcinoma, READ: Rectum adenocarcinoma, SARC: Sarcoma, SKCM: Skin Cutaneous Melanoma, STAD: Stomach adenocarcinoma, TGCT: Testicular Germ Cell Tumors, THCA: Thyroid carcinoma, THYM: Thymoma, UCEC: Uterine Corpus Endometrial Carcinoma, UCS: Uterine Carcinosarcoma, UVM: Uveal Melanoma.*

B) CDADC1 alterations in 101,480 patients from a curated set of non-redundant studies at cBioPortal. Studies with ≥50 patients and a minimum of 1% alterations in *CDADC1* were retained.

Table S1. Correlation of CDADC1 transcript levels with overall survival in cancer patients. CDADC1 expression data and survival from TCGA data was analyzed at OncoDB using 50% cut off to stratify CDADC1 expression. Long-rank test p-value, hazard ratio (HR), hazard ratio confidence intervals (CI), and sample size are indicated.

CANCER TYPE	p-value	HR	HR CI	Sample (n)
Adrenocortical carcinoma	0.92	0.96	0.46-2.02	75
Bladder urothelial carcinoma	0.91	1.02	0.76-1.36	408
Breast invasive carcinoma	0.93	0.98	0.71-1.37	1082
Cervical squamous cell carcinoma	0.76	0.93	0.58-1.48	304
Cholangiocarcinoma	0.1	2.22	0.83-5.93	36
Colon adenocarcinoma	0.57	1.14	0.72-1.83	288
Esophageal carcinoma	0.9	1.03	0.65-1.62	182
Head and neck squamous cell carcinoma	0.77	1.04	0.8-1.36	507
Kidney chromophobe	0.78	0.83	0.22-3.1	62
**Kidney clear cell carcinoma**	**3.40E-06**	**0.48**	**0.35-0.66**	**532**
Kidney renal papillary cell carcinoma	0.11	0.62	0.34-1.13	285
Liver hepatocellular carcinoma	0.02	0.66	0.47-0.94	371
**Lung adenocarcinoma**	**5.40E-03**	**0.66**	**0.49-0.89**	**499**
Lung squamous cell carcinoma	0.39	1.13	0.86-1.48	478
Diffuse large B cell lymphoma	0.81	1.18	0.29-4.74	46
Mesothelioma	0.64	0.9	0.56-1.43	83
Oropharyngeal squamous cell carcinoma	0.18	0.56	0.23-1.32	79
Ovarian serous cystadenocarcinoma	0.19	1.18	0.92-1.50	414
Pancreatic adenocarcinoma	0.12	0.72	0.48-1.09	177
Pheochromocytoma and paraganglioma	0.19	0.26	0.03-2.33	177
Prostate adenocarcinoma	0.4	0.56	0.14-2.2	494
Rectum adenocarcinoma	0.73	0.84	0.31-2.29	90
Skin cutaneous melanoma	0.05	2.12	0.99-4.54	95
Stomach adenocarcinoma	0.17	0.8	0.58-1.1	394
Testicular germ cell tumors	**0.04**	0	0-INF	133
Thyroid carcinoma	0.52	1.39	0.51-3.74	494
Uterine corpus endometrial carcinoma	0.62	0.9	0.60-1.36	542
Uterine carcinosarcoma	0.79	1.1	0.55-2.18	57
Uveal melanoma	0.17	1.87	0.76-4.6	80

Table S2 - Oligonucleotides used in this work

Oligo ID	Target or template	Sequence (5' to 3')
**gRNAs**		
**OJ1234**	Mouse Cdadc1 exon 4 gRNA1	CACC**G**CCCAGTATGTAAGTCTTCAC
**OJ1235**	Mouse Cdadc1 exon 4 gRNA1	AAACGTGAAGACTTACATACTGGG**C**
**OJ1236**	Mouse Cdadc1 exon 4 gRNA2	CACC**G**CTCTCATTAAGCATGGGTCC
**OJ1237**	Mouse Cdadc1 exon 4 gRNA2	AAACGGACCCATGCTTAATGAGAG**C**
**OJ1163**	Mouse Dctd exon 1 gRNA1	CACCGGAATGGCCCGAGTATTTCA
**OJ1164**	Mouse Dctd exon 1 gRNA1	AAACTGAAATACTCGGGCCATTCC
**OJ1165**	Mouse Dctd exon 2 gRNA2	CACC**G**AGGTCATCACTGCACCCATT
**OJ1166**	Mouse Dctd exon 2 gRNA2	AAACAATGGGTGCAGTGATGACCT**C**
**OJ928**	Human CDADC1 exon 1 gRNA1	CACCGCAAAATCTGGAGAGCGCGA
**OJ929**	Human CDADC1 exon 1 gRNA1	AAACTCGCGCTCTCCAGATTTTGC
**OJ930**	Human CDADC1 exon 1 gRNA2	CACC**G**CTGGGTGCTGACTGACCGCC
**OJ931**	Human CDADC1 exon 1 gRNA2	AAACGGCGGTCAGTCAGCACCCAG**C**
**OJ936**	Human CDADC1 exon 4 gRNA1	CACCGTAAATCTTCACTAGAACAG
**OJ937**	Human CDADC1 exon 4 gRNA1	AAACCTGTTCTAGTGAAGATTTAC
**OJ938**	Human CDADC1 exon 4 gRNA2	CACC**G**AGATTGCTCTTATTAAACAT
**OJ939**	Human CDADC1 exon 4 gRNA2	AAACATGTTTAATAAGAGCAATCT**C**
**OJ932**	Human CDADC1 exon 5 gRNA1	CACC**G**CAGCAGGCCAGTATGAAATT
**OJ933**	Human CDADC1 exon 5 gRNA1	AAACAATTTCATACTGGCCTGCTG**C**
**OJ934**	Human CDADC1 exon 5 gRNA2	CACC**G**AGAAATAAGTTTGCTTACGG
**OJ935**	Human CDADC1 exon 5 gRNA2	AAACCCGTAAGCAAACTTATTTCT**C**
**OJ1147**	Human DCTD exon 2 gRNA1	CACC**G**CAAGAAACGGGACGACTATT
**OJ1148**	Human DCTD exon 2 gRNA1	AAACAATAGTCGTCCCGTTTCTTG**C**
**OJ1149**	Human DCTD exon 3 gRNA2	CACC**G**ATTGTCGGGATTGGGTACAA
**OJ1150**	Human DCTD exon 3 gRNA2	AAACTTGTACCCAATCCCGACAAT**C**
**OJ3598**	human SAMHD1 gRNA1	cacc**g**AAACGAGACTCATCAAGACA
**OJ3599**	human SAMHD1 gRNA1	aaacTGTCTTGATGAGTCTCGTTTc
**OJ3420**	human DCTPP1 gRNA1	CACC**G**CTTACATGTCCTCGAGCGT
**OJ3421**	human DCTPP1 gRNA1	AAACACGCTCGAGGACATGTAAGC
**OJ3422**	human DCTPP1 gRNA2	CACC**G**TTCCGCAGCAAACTCAGCA
**OJ3423**	human DCTPP1 gRNA2	AAACTGCTGAGTTTGCTGCGGAAC
**Genotyping primers**		
**OJ1228**	Mouse Cdadc1 exon 4 deletion fwd	ATTAGGTGAAGAAAACTGGTCTTG
**OJ1229**	Mouse Cdadc1 exon 4 deletion rev	CACCTTGTCCATACTCACCATT
**OJ1157**	Mouse Dctd exon1 and 2 deletion fwd	TGCTCTCTTTGAAGTCTAGGGC
**OJ1158**	Mouse Dctd exon1 and 2 deletion rev	AGGCACCTTACCATAAGGATATTT
**OJ1028**	Human CDADC1 exon 4 deletion fwd	GTAAAGAGAACTGGTCTTGTGGTGG
**OJ1029**	Human CDADC1 exon 4 deletion rev	CCAACAAACCCTTTTCTGCACTTACC
**OJ1030**	Human CDADC1 exon 5 deletion fwd	GGGTTCTAGATATACTGCAATGATAGGGTGG
**OJ1031**	Human CDADC1 exon 5 deletion rev	GGTCTCCTCTACAAACTGCACCATA
**OJ2324**	Human CDADC1 exon 4 deletion fwd	GCCTAGGTTTAGAAAATTTCTGGCCTAAAGT
**OJ2325**	Human CDADC1 exon 4 deletion rev	TCCCACCTCATTATCTCAAGTAGTGGG
**OJ1151**	Human DCTD exon 2 and 3 deletion fwd	CGGGTATTTGGTGTCCAGCT
**OJ1152**	Human DCTD exon 2 and 3 deletion rev	TGTGGTTGGGTTGAACTGGA
**OJ2323**	Human DCTD exon 2 and 3 deletion fwd	CGGGTATTTGGTGTCCAGCT
**OJ3602**	Human SAMHD1 exon 2 fwd	AAAAGTGGTACTTGGCACAATCC
**OJ3603**	Human SAMHD1 exon 2 rev	AGGCAAGGGATTCTGCTGTTTTA
**OJ3426**	Human DCTPP1 exon 1 fwd	TGGGTTTTCCGTGAAGTCGC
**OJ3427**	Human DCTPP1 exon 1 rev	TAGGCTGAAGGGCGTTCCTA
**OJ3479**	Human DCTPP1 exon 2 fwd	CCTCTAACCCGGAGACTTGG
**OJ3480**	Human DCTPP1 exon 2 rev	ACTGCTTCTTCCTAGACGGG
**RT-qPCR**		
**OJ849**	Mouse Cdadc1 exon 4	AACCATGTTCTGCTTGTTTGAA
**OJ850**	Mouse Cdadc1 exon 5	TGGGTCAGAAGGCCAGTAAG
**OJ897**	mouse Actin	CTCTGGCTCCTAGCACCATGAAGA
**OJ898**	mouse Actin	GTAAAACGCAGCTCAGTAACAGTCCG
**OJ2777**	human DCTPP1	CCTGAGGCCTCCACTTCTTG
**OJ2778**	human DCTPP1	TCCCTGGCCATCCAGGAATA
**OJ1076**	human RPS15	GAAGTGGAGCAGAAGAAGA
**OJ1077**	human RPS15	CTGCATCAGTTGCTCATAG
**OJ1305**	human DUT	GAGAAAGCTGTTGTGAAAACGGA
**OJ1306**	human DUT	CTATGACACCAGCTCCTACATCA
**OJ1012**	human Actin	TCCCTGGAGAAGAGCTACGA
**OJ1013**	human Actin	AGCACTGTGTTGGCGTACAG
**Cloning**		
**OJ1000**	Human CDADC1 v2	CCGTTGCCTTACAGTTGCTG
**OJ1001**	Human CDADC1 v2	TTGGATGGTCGTGCGTGTAA
**OJ1002**	Human CDADC1 v1	GTTCTTGAGGTTCCCCTGCA
**OJ1125**	Human CDADC1	GATACATCATACATGCGGCGCAGAATGCCTTGACATT
**OJ1126**	Human CDADC1	AATGTCAAGGCATTCTGCGCCGCATGTATGATGTATC
**OJ3591**	Human CDADC1 v1 E coli codon opt	CATTCACGCGgcgCAGAACGCGC
**OJ3592**	Human CDADC1 v1 E coli codon opt	ATATAACGGAACTTACGGATTTCACGATCC
**OJ1032**	Human DCTD	ggatccGAATGGTGGGCGGGGG
**OJ1033**	Human DCTD	ggtaccTCACTGAAGCTTTTGACTCGGT
**OJ2168**	hCDADC1	atatACTAGTATGAAAGAAGCTGGGCAGATGC
**OJ1419**	hCDADC1	CCGCGGATGAAAGAAGCTGGGCAGATGC
**OJ1420**	hCDADC1	GCGGCCGCTTAGTGGATTCCGAGGCGCAG
**OJ1902**	Ugi	GCGttaattaaATGACAAATTTATCTGACATC
**OJ1903**	Ugi	GCGttaattaaTTATAACATTTTAATTTTATTTTCTCCATTAC
**OJ96**	UNG substrate	**bio**-ATTATTATTATTCCG**U**GGATTTATTTATTTATTTATTTATTT-**fitc**
**OJ97**	UNG substrate	AAATAAATAAATAAATAAATAAATCC**G**CGGAATAATAATAAT
**shRNA**		
**OJ2849**	human DUT	CCGGTGGTTCCACTGGAAAGAATTACTCGAGTAATTCTTTCCAGTGGAACCATTTTTG
**OJ2850**	human DUT	AATTCAAAAATGGTTCCACTGGAAAGAATTACTCGAGTAATTCTTTCCAGTGGAACCA
**OJ2851**	mouse DUT	CCGGCGGATTTCTTATCCAGACTTACTCGAGTAAGTCTGGATAAGAAATCCGTTTTTG
**OJ2852**	mouse DUT	AATTCAAAAACGGATTTCTTATCCAGACTTACTCGAGTAAGTCTGGATAAGAAATCCG
**OJ2853**	mouse DUT	CCGGGCTGGAAGTATCTCGCTGTTTCTCGAGAAACAGCGAGATACTTCCAGCTTTTTG
**OJ2854**	mouse DUT	AATTCAAAAAGCTGGAAGTATCTCGCTGTTTCTCGAGAAACAGCGAGATACTTCCAGC

Table S3- Cell clones generated by CRISPR/Cas9 in different cell lines

Parental cell line	Gene	Clone ID	Targeted Exons	Alleles	Genomic Indels (bp)	Protein deletion (# aa)	Del or Frameshift (last correct aa)	Stop codon # * length of protein when in frame del
HEK293T	*CDADC1*	F5	exon 4	Both	del 45	15 aa	del Ser 104 - Lys 117	499*
HEK293T	*CDADC1*	F2	exon 5	allele 1	del 45		Ile 149	156
				allele 2	del 38	16 aa	del Asn 147 - Thr 162	498*
HEK293T	*CDADC1*	D3	exon 4	Both	del 42		His 102	103
HEK293T	*CDADC1*	F7	exon 4	Both	del 53		Leu 101	108
HEK293T	*DCTD*	#24	exons 2-3	allele 1	del 526		Asp 11	46
				allele 2	(ins +2) del 528		Asp 11	46
HEK293T	*DCTD*	#35	exons 2-3	Both	del 526		Asp 11	46
HEK293T CDADC1 KO F7	*DCTD*	D5	exons 2-3	allele 1	del 526		Asp 11	46
				allele 2	del 525	40 aa	del Leu 13 - Tyr 53	138
HEK293T CDADC1 KO F7	*DCTD*	C7	exons 2-3	Both	(ins +123) del 527		Asp 10	61
					del 526		Asp 11	46
HEK293T	*SAMHD1*	#6	exon 2	Both	del 5		Pro 79	80
HEK293T	*SAMHD1*	#8	exon 2	Both	del 5		Pro 79	80
HEK293T CDADC1 KO F5	*SAMHD1*	dKO #1	exon 2	Both	del 2		Pro 79	81
HEK293T CDADC1 KO F5	*SAMHD1*	dKO #6	exon 2	Both	del 5		Pro 79	80
HEK293T	*DCTPP1*	#1	exon 1	Both	del 4		Thr 30	105
HEK293T	*DCTPP1*	#7	exon 2	Both	ins +1		Ala 39	40
HEK293T CDADC1 KO F5	*DCTPP1*	dKO #3	exon 1	Both	del 7		Thr 30	104
HEK293T CDADC1 KO F5	*DCTPP1*	dKO #12	exon 2	Both	ins +1		Ala 39	40
CH12	*Cdadc1*	B8	exon 4	allele 1	del 64		Ile 99	102
				allele 2	del 108	36 aa	del Ile 99 - Pro 134	499*
CH12	*Cdadc1*	D2	exon 1	Both	del 15	5 aa	del Gly 12 - Glu 16	509*
CH12	*Dctd*	#2	exons 1-2	Both	del ~ 300			
CH12	*Dctd*	#19	exons 1-2	Both	del ~ 300			
CH12 Cdadc1 B8	*Dctd*	B8C6	exons 1-2	Both	del ~ 300			
CH12 Cdadc1 B8	*Dctd*	B8D7	exons 1-2	Both	del ~ 300			
MOLT4	*CDADC1*	E7	exon 4	Both	del ~40			
MOLT4	*CDADC1*	F5	exon 5	Both	del ~40			
MOLT4	*CDADC1*	MCG	exon 4	Both	del 43		His 102	108
MOLT4	*CDADC1*	MCB	exon 4	Both	del 43		His 102	108
JURKAT	*CDADC1*	JC-5	exon 4	Both	del 47		His 102	110
JURKAT	*CDADC1*	JC-6	exon 4	Both	del 47		His 102	110
THP1	*CDADC1*	TC25	exon 4	Both	del 42	14	del Ser 104 - Lys 117	500*
THP1	*CDADC1*	TC56	exon 4	Both	del 43		His 102	108
THP1	*CDADC1*	TC60	exon 4	allele 1	del >40			
				allele 2	del~40			
THP1	*CDADC1*	TC69	exon 4	allele 1	del 42	14	del Ser 104 - Lys 117	500*
				allele 2	del 235		Glu 84	100
THP1	*DCTD*	TD01	exons 2-3	Both	del ~ 800			
THP1	*DCTD*	TD05	exons 2-3	Both	del ~ 800			
K562	*CDADC1*	#1	exon 4	Both	del 43		His 102	108
K562	*CDADC1*	#5	exon 4	Both	del 43		His 102	108
K562	*CDADC1*	#11	exon 4	Both	del 43		His 102	108
A549	CDADC1	A19	exon 4	Both	del 43		His 102	108
A549	*CDADC1*	A33	exon 4	Both	del~40			
A549	*CDADC1*	A57	exon 4	Both	del 2		Ile 116	125
H1299	*CDADC1*	H1	exon 4	Both	del 43		His 102	108
H1299	*CDADC1*	H15	exon 4	Both	del 44		Cys 103	111
H1299	*CDADC1*	H25	exon 4	Both	del 15		Leu 115	117
KPC	*Cdadc1*	E2	exon 4	Both	del 41	14 aa	Ser 106	128
KPC	*Cdadc1*	F4	exon 4	Both	del 41	14 aa	Ser 106	128
KP4	*CDADC1*	K2	exon 4	Both	del 43		His 102	108
KP4	*CDADC1*	K8	exon 4	Both	del 43		His 102	108
KP4	*CDADC1*	K19	exon 4	Both	del 43		His 102	108

Dataset S1 (separate file). The table provides the CRANKS scores for all genes in the genome wide CRISPR/Cas0 screen made in NALM-6 cells grown in gemcitabine. A positive score indicates enhanced growth (i.e. more resistance), and a negative score indicates reduced growth (i.e., increased sensitivity) for each corresponding gene KO.

SI References

1. K.-J. Shin, *et al.*, A single lentiviral vector platform for microRNA-based conditional RNA interference and coordinated transgene expression. *Proc Natl Acad Sci U S A* **103**, 13759–64 (2006).
2. J. M. Di Noia, M. S. Neuberger, Altering the pathway of immunoglobulin hypermutation by inhibiting uracil-DNA glycosylase. *Nature* **419**, 43–8 (2002).
3. B. Weiss, L. Wang, De novo synthesis of thymidylate via deoxycytidine in dcd (dCTP deaminase) mutants of Escherichia coli. *J Bacteriol* **176**, 2194–2199 (1994).
4. G. W. Camiener, C. G. Smith, Studies of the enzymatic deamination of cytosine arabinoside. I. Enzyme distribution and species specificity. *Biochem Pharmacol* **14**, 1405–16 (1965).
5. T. P. WANG, H. Z. SABLE, J. O. LAMPEN, Enzymatic deamination of cytosine nucleosides. *J Biol Chem* **184**, 17–28 (1950).
6. A. Awasthi, D. HM, M. B. Sathyanarayana, Novel Validated UV Spectroscopic Method for Routine Analysis of Decitabine Drug Substance. *Indian Journal of Pharmaceutical Education and Research* **54**, s667–s670 (2020).
7. Y. Zhang, *et al.*, Chloroviruses Encode a Bifunctional dCMP-dCTP Deaminase That Produces Two Key Intermediates in dTTP Formation. *J Virol* **81**, 7662–7671 (2007).
8. E. Murakami, *et al.*, The mechanism of action of β-D-2′-deoxy-2′-fluoro- 2′-C-methylcytidine involves a second metabolic pathway leading to β-D-2′-deoxy-2′-fluoro-2′-C-methyluridine 5′-triphosphate, a potent inhibitor of the hepatitis C virus RNA-dependent RNA polymerase. *Antimicrob Agents Chemother* **52**, 458–464 (2008).
9. M. Nakamura, *et al.*, High frequency class switching of an IgM+ B lymphoma clone CH12F3 to IgA+ cells. *Int Immunol* **8**, 193–201 (1996).
10. G. Henneré, *et al.*, Liquid chromatography–tandem mass spectrometry assays for intracellular deoxyribonucleotide triphosphate competitors of nucleoside antiretrovirals. *Journal of Chromatography B* **789**, 273–281 (2003).
11. K. Normandin, *et al.*, Genetic enhancers of partial PLK1 inhibition reveal hypersensitivity to kinetochore perturbations. *PLoS Genet* **19**, e1010903 (2023).
12. T. Bertomeu, *et al.*, A High-Resolution Genome-Wide CRISPR/Cas9 Viability Screen Reveals Structural Features and Contextual Diversity of the Human Cell-Essential Proteome. *Mol Cell Biol* **38** (2018).
13. K. Tamura, *et al.*, MEGA5: Molecular evolutionary genetics analysis using maximum likelihood, evolutionary distance, and maximum parsimony methods. *Mol Biol Evol* **28**, 2731–2739 (2011).
14. M. Nei, S. Kumar, *Molecular Evolution and Phylogenetics* (Oxford University Press, 2000).
15. J. Felsenstein, CONFIDENCE LIMITS ON PHYLOGENIES: AN APPROACH USING THE BOOTSTRAP. *Evolution* **39**, 783–791 (1985).
16. G. E. Crooks, G. Hon, J.-M. Chandonia, S. E. Brenner, WebLogo: a sequence logo generator. *Genome Res* **14**, 1188–90 (2004).
17. M. Uhlén, *et al.*, Tissue-based map of the human proteome. *Science (1979)* **347** (2015).
18. E. Cerami, *et al.*, The cBio cancer genomics portal: an open platform for exploring multidimensional cancer genomics data. *Cancer Discov* **2**, 401–404 (2012).
19. Z. Tang, *et al.*, GEPIA: A web server for cancer and normal gene expression profiling and interactive analyses. *Nucleic Acids Res* **45**, W98–W102 (2017).
20. G. Tang, M. Cho, X. Wang, OncoDB: An interactive online database for analysis of gene expression and viral infection in cancer. *Nucleic Acids Res* **50**, D1334–D1339 (2022).

21. T. Haferlach, *et al.*, Clinical utility of microarray-based gene expression profiling in the diagnosis and subclassification of leukemia: report from the International Microarray Innovations in Leukemia Study Group. *J Clin Oncol* **28**, 2529–2537 (2010).
22. S. Chen, *et al.*, A genomic mutational constraint map using variation in 76,156 human genomes. *Nature* **625**, 92–100 (2024).
